# Molecular Simulations Investigation of Deep Eutectic Solvent Induced Structural and Interfacial Behavior in Surfactant-Enhanced Oil Recovery Systems

**DOI:** 10.1021/acsomega.6c06556

**Published:** 2026-10-01

**Authors:** Subodh Sinha, Abhishek Kumar Gupta

**Affiliations:** Department of Chemical Engineering, 30133 Indian Institute of Technology Kharagpur, Kharagpur, West Bengal 721302, India

## Abstract

Chemically enhanced oil recovery (c-EOR) relies heavily on surfactant-induced reduction of oil–water interfacial tension; however, the molecular-level role of deep eutectic solvent (DES) chemistry in surfactant-assisted interfacial stabilization remains poorly understood. In this work, atomistic molecular dynamics simulations were employed to investigate the interfacial behavior of choline chloride-based deep eutectic solvents (DESs) in combination with the anionic surfactant sodium dodecylbenzenesulfonate (SDBS) at the oil–water interface. Two DES systems, choline chloride–ethylene glycol (ChCl/EG) and choline chloride–cresol (ChCl/cresol), were examined over a concentration range of 10–30 wt %. Density distribution, hydrogen-bonding, radial distribution function, and RDF-derived excess entropy analyses reveal that the surfactant remains stably adsorbed at the interface, while DES molecules predominantly occupy the aqueous phase and progressively enrich the interfacial region with increasing concentration. Both DES systems significantly reduce interfacial tension, with the IFT decreasing from approximately 12.4 mN/m in the DES-free system to 8.47 mN/m for ChCl/EG and 7.23 mN/m for ChCl/cresol at 30 wt % DES concentration. The ChCl/EG system exhibits a gradual reduction in interfacial tension driven by cooperative hydrogen-bond-mediated restructuring of the aqueous phase and relatively uniform intermolecular ordering. In contrast, the ChCl/cresol system produces stronger interfacial effects at higher concentrations due to the greater interfacial affinity and localized accumulation of cresol molecules near the oil–water boundary. Hydrogen-bonding and excess entropy analyses further demonstrate that the EG-based DES promotes relatively diffuse and cooperative aqueous structuring, whereas the cresol-based DES induces more localized and concentration-dependent intermolecular organization near the interface. These findings demonstrate that DES chemistry plays a critical role in governing intermolecular organization, hydration structure, and interfacial stabilization in surfactant-assisted c-EOR systems. The molecular-level insights obtained in this study provide useful guidelines for the rational design of DES–surfactant formulations for EOR applications.

## Graphical abstract

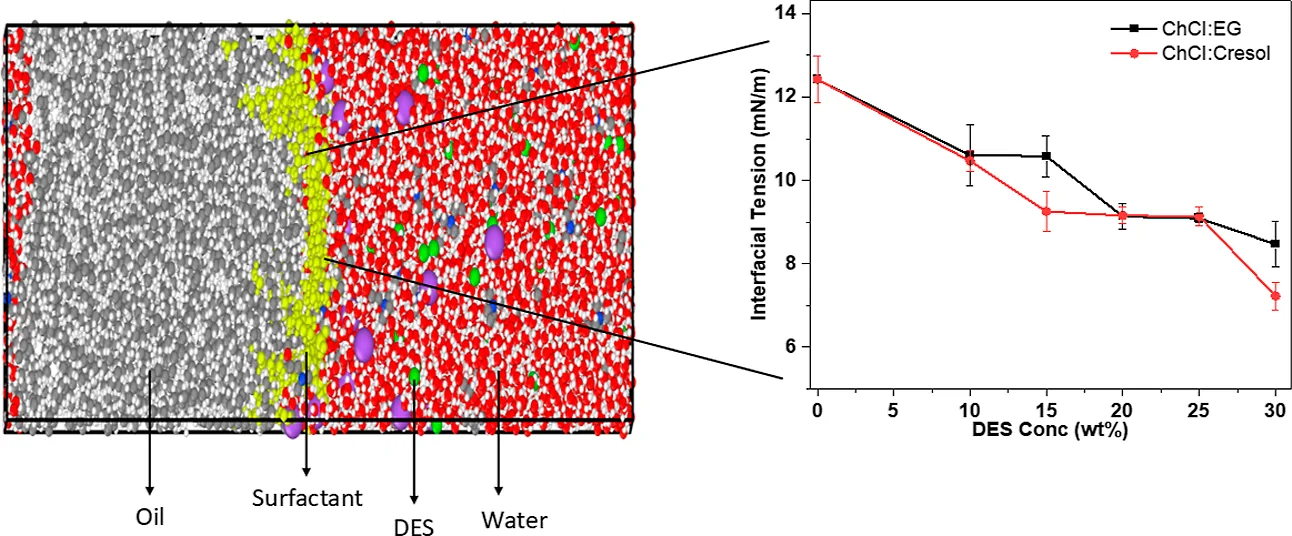

## 1 Introduction

As global energy demand continues to increase due to rapid population growth, industrialization, and expanding infrastructure, the strain on existing petroleum reserves has intensified significantly.^1^ Despite remarkable progress in renewable energy technologies, their widespread replacement of fossil fuels remains constrained by several technical and economic challenges, including intermittency, inadequate large-scale energy storage systems, and high implementation costs.^2,3^ Consequently, crude oil continues to play a dominant role in meeting the global energy requirements. However, conventional primary and secondary oil recovery techniques can extract only a limited portion of the original oil in place, leaving a substantial amount of hydrocarbons trapped within reservoir pores due to capillary and viscous forces.^4^ In the face of growing energy demand and the progressive depletion of easily accessible oil reserves, enhanced oil recovery (EOR) technologies have become increasingly important for improving reservoir productivity and extending the lifespan of mature oil fields. Among various EOR methods, chemically enhanced oil recovery (c-EOR) has emerged as an effective and economically attractive approach for mobilizing residual oil and enhancing overall recovery efficiency.^5^

In most hydrocarbon reservoirs, oil recovery during the primary and secondary production stages is inherently limited. As reservoir pressure declines and conventional water flooding becomes less effective, a significant portion of the oil remains trapped within the pore spaces of the rock matrix due to capillary forces and unfavorable fluid–rock interactions.^6^ This unrecovered fraction, commonly referred to as residual oil, may account for nearly 30–60% of the original oil in place.^7^ With conventional oil exploration becoming increasingly challenging and expensive, maximizing recovery from mature and depleted reservoirs has emerged as a strategic priority for the petroleum industry. In this context, EOR techniques play a crucial role in extending reservoir productivity, improving hydrocarbon recovery efficiency, and contributing to the growing global energy demand.^8^ Surfactant-based EOR is advantageous because it reduces interfacial tension (IFT), alters the wettability of the rock, promotes emulsification, and enhances the efficiency of microscopic displacement.^8,9^ Three surfactants, namely, sodium dodecyl sulfate, sodium oleate, and APG0810, were used to reduce the interfacial tension of the water–oil interface, alter the wettability and adsorption on rock surfaces using combined experiments and molecular dynamics (MD) simulations.^10^ It was investigated that the greatest interfacial tension reduction occurs in the case of sodium oleate from 52.4 mN/M to 0.0027 mN/M with SO being the optimal surfactant.^10^ The sodium dodecyl benzenesulfonate (SDBS) micelle interactions with model additives, i.e., anisole and 1-hexene in the crude oil was investigated using experimental methods employing light scattering, UV-vis, H-NMR, TEM, and small angle neutron scattering. It was demonstrated that the SDBS micelles become swollen with increasing concentration of additives with 1-hexene residing in the micelle core, while the 1-anisole facilitates the weakening of electrostatic repulsion among SDBS head-groups.^12^ The effect of different surfactants such as SDSn, SDS, SDBS, and AES on the water–oil interfacial tension has been measured experimentally utilizing tensiometry, and MD simulations have been performed to quantify interfacial thickness, interfacial energy, and surfactant-water structure wherein it was investigated that the addition of benzene ring and oxyethyl group to the surfactant enhances the surfactant polarity, thereby leading to a stronger interaction between surfactant headgroup and water molecules.^13^ A reduction in interfacial tension (IFT) decreases capillary forces, thereby enhancing the mobility and recovery of trapped oil. Despite its effectiveness, surfactant flooding faces several challenges, including high chemical costs, surfactant adsorption onto reservoir rock surfaces, limited sweep efficiency, and potential environmental concerns.^14^ These limitations have motivated researchers to explore alternative chemical systems and additives that can improve the efficiency, economic viability, and sustainability of chemically enhanced oil recovery (c-EOR) processes. In recent years, significant attention has been directed toward reducing surfactant consumption, optimizing chemical formulations, and identifying stable chemical agents capable of withstanding harsh reservoir conditions, including high salinity and elevated temperatures.^15^ Deep eutectic solvents (DESs) are a promising class of solvents that can withstand harsh reservoir conditions, exhibit lower adsorption on reservoir rock surfaces, and demonstrate strong potential for heavy oil recovery applications. Additionally, DESs are relatively inexpensive, environmentally sustainable, and easy to synthesize.^16^ DESs are eutectic mixtures composed of a hydrogen bond donor (HBD) and a hydrogen bond acceptor, which, when combined in specific molar ratios, form liquids with melting points significantly lower than those of the individual components.^17,18^

Recent experimental and molecular simulation studies have investigated various classes of DESs as functional additives for EOR applications. Among them, hydrophilic DESs such as choline chloride–urea (ChCl/urea) and choline chloride–ethylene glycol (ChCl/EG) have attracted considerable attention due to their strong hydrogen-bonding networks, high water miscibility, and thermal stability under harsh reservoir conditions. Experimental studies have demonstrated that these DES systems can significantly reduce oil–water interfacial tension (IFT), maintain stability under high-salinity and high-temperature conditions, and alter reservoir wettability toward a more water-wet state, thereby enhancing oil displacement efficiency.^19,20^ At the same time, hydrophobic DESs, such as menthol–salicylic acid, have attracted considerable attention because of their strong affinity toward nonpolar crude oil components. MD simulation studies on ChCl/urea, ChCl/EG, and menthol–salicylic acid systems have demonstrated that DES molecules preferentially adsorb at oil–water and solid–fluid interfaces. At these interfaces, DES molecules reorganize to disrupt cohesive oil–oil interactions and reduce adhesive forces between hydrocarbons and mineral surfaces, including silica and calcite. Such interfacial adsorption facilitates the detachment of trapped oil, reduces capillary trapping, and enhances the mobilization of residual hydrocarbons within porous reservoir rocks.^21,22^ Experimental investigations and atomistic simulation studies collectively demonstrate that the EOR performance of DESs is governed by their physicochemical characteristics, hydrogen-bonding interactions, and interfacial behavior.^19–22^

Based on these findings, the integration of DESs with conventional surfactants—rather than their use as complete substitutes—has emerged as a promising strategy for EOR. The central premise is that DESs can independently reduce interfacial tension and/or modify surface wettability, thereby lowering the amount of surfactant required while maintaining comparable oil recovery efficiency. This synergistic approach addresses two major limitations of conventional surfactant flooding: high chemical costs and environmental concerns. In addition, DES–surfactant hybrid systems have demonstrated superior performance under harsh reservoir conditions, particularly at high salinity and elevated temperatures, where DESs enhance surfactant stability, interfacial activity, and overall EOR effectiveness.^23^ Despite growing interest in DESs for EOR, the molecular-level role of DES chemistry in governing surfactant-assisted interfacial behavior remains weakly understood. Most previous studies have primarily focused on macroscopic properties such as interfacial tension reduction and wettability alteration, while limited attention has been given to how different hydrogen bond donors influence surfactant adsorption, water structuring, and interfacial stabilization mechanisms. Furthermore, the combined use of DESs and surfactants introduces complex hydrogen-bonding and electrostatic interactions that cannot be fully resolved experimentally. Therefore, atomistic MD simulations are essential to establish direct relationships between DES chemistry, interfacial organization, and thermodynamic behavior in surfactant-based EOR systems. In the present work, the effect of DESs on the interfacial structure and dynamics of surfactant-based c-EOR systems is investigated. The synergistic interactions between DESs and surfactants are examined at the molecular level using atomistic simulations to elucidate the interfacial structure, interfacial behavior, and thermodynamic properties at the oil–water interface. The molecular-level insights obtained from this study are expected to contribute significantly toward the rational design of advanced c-EOR formulations.

## 2 Computational Methodology

### 2.1 Simulation Setup

The system considered in this study was designed to represent a simplified model of hydrocarbon–water interactions commonly encountered under reservoir conditions. The oil phase consisted of dodecane (C_12_H_26_) and octane (C_8_H_18_), which are representative components of the light hydrocarbon fraction present in crude oil.^24^ Real light crude oil is compositionally complex, containing a broad range of paraffins, naphthenes, aromatics, resins, and asphaltenes. In the present study, the dodecane–octane binary mixture was intentionally employed as a simplified model representing the predominantly nonpolar hydrocarbon fraction of light crude oil. This simplified representation allows the effects of DES incorporation and SDBS adsorption at the oil–water interface to be examined without introducing the additional molecular complexity associated with heavier and more chemically diverse crude-oil components. The aqueous phase was modeled using the extended simple point charge (SPC/E) water model. The SPC/E model was chosen because it reliably reproduces the bulk density, dielectric response, and self-diffusion coefficient of water at 298 K. Furthermore, it offers superior computational efficiency and excellent phase-coexistence behavior when integrated with the OPLS-AA framework for long-time scale multi-component liquid interfaces compared to more resource-intensive multi-site models.^25,26^

Two different DESs, namely, choline chloride–ethylene glycol (ChCl/EG) and choline chloride–cresol (ChCl/cresol), were considered in the present study. To investigate the effect of surfactants, sodium dodecyl benzenesulfonate (SDBS), an anionic surfactant, was employed as the model surfactant system. SDBS comprises a hydrophobic dodecyl alkyl chain linked to a benzene ring and a hydrophilic sulfonate head group, enabling strong interfacial activity and pronounced electrostatic interactions in aqueous environments. The surfactant was selected because of its well-established effectiveness in reducing oil–water interfacial tension and its widespread application in c-EOR studies.^27^ The number of water and surfactant molecules was kept constant across the concentration series so that DES content was the only compositional variable. This approach was adopted intentionally to isolate the effect of DES chemistry and concentration while avoiding confounding effects arising from changes in surfactant areal density or water content. The chemical structures of all the compounds considered in this work are presented in Figure 1.

**Figure 1:**
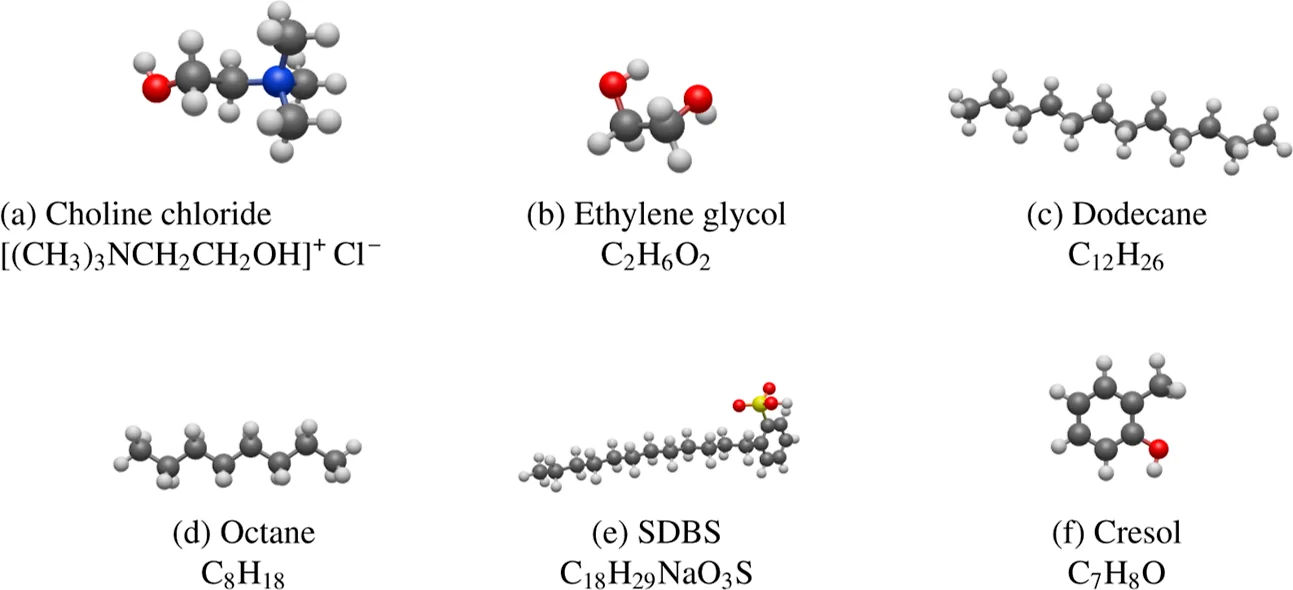
Molecular structures and molecular formulas of the surfactant, DES components, and hydrocarbon molecules considered in this study.

The geometry optimization and partial atomic charge calculations for all molecules considered in this study were performed using density functional theory. All quantum chemical calculations were carried out using the Gaussian 16 software package.^28,29^ The molecular geometries were initially optimized to obtain energetically stable conformations prior to subsequent electronic structure calculations. The B3LYP hybrid exchange–correlation functional in conjunction with the 6-31G­(d,p) basis set was employed for both geometry optimization and partial charge calculations. The partial charges were calculated using the CHELPG charge scheme.^30^

Simulations were performed at different DES concentrations. The number of molecules used at different concentrations is shown in Table 1. The initial simulation box was constructed using PACKMOL.^31^ The system has a box size of 50 × 50 × 140 Å. An explicit DES–water–oil interfacial system was constructed along the *z*-axis, with the surfactant molecules initially positioned at the interface. Periodic boundary conditions were applied in all three spatial directions.

**Table 1:** Details of the Simulation System Used in the Study

DES concentration	ChCl/cresol		ChCl/EG		water molecules	surfactant molecules
	choline chloride	cresol	choline chloride	ethylene glycol		
10%	17	34	23	46	3000	15
15%	27	54	38	76	3000	15
20%	38	76	52	104	3000	15
25%	50	100	70	140	3000	15
30%	65	130	89	178	3000	15

### 2.2 Simulation Methodology

MD simulations were carried out using the LAMMPS MD engine. The anionic surfactant and DES (ChCl-EG and ChCl-cresol) were modeled using the OPLS-AA force field.^32^ The force field parameters (bonded and non-bonded) of DES systems constituting ChCl/EG and ChCl/cresol are displayed in Tables S1–S8 (Supporting Information section). The bond, angle, and dihedral parameters were adopted from the OPLS-AA force field based on the corresponding atom types and were not further re-optimized against quantum-mechanical vibrational frequencies or torsional–energy profiles. Only the partial atomic charges were replaced with CHELPG charges obtained from B3LYP/6-31G­(d,p) calculations. The water molecules were modeled using the SPC/E model.^25^ The equations of motion were integrated using the velocity–Verlet algorithm. The simulation protocol began with energy minimization using the steepest descent^33^ and conjugate gradient algorithms to remove atomic overlaps and relax the initial configuration. Following energy minimization, the systems were equilibrated under the *NVT* ensemble for 100 ps at 298 K, stabilizing the system temperature. This was followed by *NPT* equilibration for 300 ps at *P* = 1 atm and 298 K to achieve the correct system density and pressure equilibrium.

The temperature was controlled using a Nosé–Hoover thermostat with a relaxation time (*T*_damp_) of 100 fs. For the *NPT* simulations, pressure was controlled using a Nosé–Hoover barostat with a relaxation time (*P*_damp_) of 1 ps.^34^ A timestep of 2 fs was employed throughout the simulations. Electrostatic interactions were treated using the particle–particle particle-mesh (PPPM) method with a real-space cutoff of 10 Å with an accuracy of 10^–5^. In contrast, van der Waals interactions were modeled using the Lennard–Jones potential with geometric mixing rules. The total potential energy of the system is expressed as the sum of bonded and non-bonded interactions

1 $$E_{total}=E_{bond}+E_{angle}+E_{dihedral}+E_{non‐bonded}$$

The bonded interactions include bond stretching, angle bending, and torsional contributions and are written as

2 $$E_{bond}=\sum_{bonds}k_{b}{\left(r-r_{0}\right)}^{2}$$

3 $$E_{angle}=\sum_{angles}k_{\theta }{\left(\theta -\theta_{0}\right)}^{2}$$

4 $$E_{dihedral}=\sum_{dihedrals}\left[\frac{V_{1}}{2}\left(1+\cos\phi \right)+\frac{V_{2}}{2}\left(1-\cos2\phi \right)+\frac{V_{3}}{2}\left(1+\cos3\phi \right)+\frac{V_{4}}{2}\left(1-\cos4\phi \right)\right]$$

The non-bonded interactions consist of Lennard–Jones and Coulombic electrostatic interactions

5 $$E_{non‐bonded}=\sum_{i<j}\left[4\varepsilon_{ij}\left({\left(\frac{\sigma_{ij}}{r_{ij}}\right)}^{12}-{\left(\frac{\sigma_{ij}}{r_{ij}}\right)}^{6}\right)+\frac{q_{i}q_{j}}{4\pi \varepsilon_{0}r_{ij}}\right]$$

Water molecules were constrained using the SHAKE algorithm^35^ to preserve bond lengths and angles, and neighbor lists were updated every time step to ensure accurate force evaluation. The initial conformation of the system is shown in Figure 2. After equilibration, a production run of 50 ns was performed under the isothermal–isobaric (*NPT*) ensemble, during which the thermodynamic and structural data were collected. To assess the stability of the 50 ns production simulations, the time evolution of system density and potential energy was examined for representative DES concentrations for both ChCl/EG and ChCl/cresol systems. As shown in Figure S1 of the Supporting Information, both density and potential energy fluctuate around stable mean values without any systematic drift over the 50 ns production period. These results support the adequacy of the selected 50 ns production length for the present analysis.

**Figure 2:**
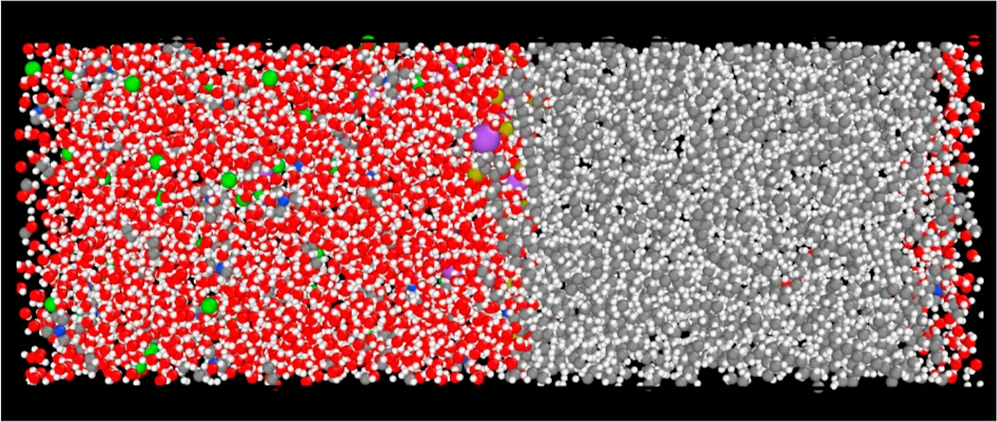
Representative initial conformational snapshot of the surfactant–DES–oil–water system at 10 wt % DES concentration, illustrating the spatial distribution of the oil phase, aqueous phase, SDBS surfactant molecules, and DES components prior to MD simulations. The color code indicates oxygen (red), hydrogen (white), carbon (gray), chlorine (purple), nitrogen (green), and sulfur (yellow).

To assess the compatibility of the OPLS-AA force field with the CHELPG-derived partial atomic charges, independent bulk simulations of choline chloride/ethylene glycol (1:2) and choline chloride/cresol (1:2) were performed at 298 K and 1 bar. The simulated densities and viscosities of both DES systems were compared to the corresponding experimental values. The density and viscosity of both DES systems (molar ratio 1:2) matches closely to the experimental values, as shown in Table 2.^36^ Although the bulk densities are slightly underestimated, the CHELPG-derived charges combined with the OPLS-AA force field reproduce stable liquid phases and provide a reasonable representation of the DES systems for investigating the relative structural and interfacial trends reported in this work. Bonded parameters (bond, angle, and dihedral) were transferred directly from the OPLS-AA library and were not reoptimized against DFT-derived data; this hybrid approach is consistent with prior DES simulation studies.^38^

**Table 2:** Comparison of the Bulk Density and Viscosity of the DES Systems at 298 K and 1 atm

DES system	density (kg m^–3^)		viscosity (mPa s)	
	experiment	MD simulation	experiment	MD simulation
ChCl/EG	1080 ^36^	995	36 ^36^	38
ChCl/cresol	1071.9 ^37^	987	133.68 ^37^	132.7

## 3 Results and Discussion

### 3.1 Density

Density distribution profiles along the *z*-direction were calculated to examine the spatial arrangement of surfactant and DES molecules across the oil–water interface. The analysis provides molecular-level insight into interfacial thickness, adsorption behavior, film formation, and the solvation characteristics of SDBS and DES molecules at the interface. These profiles also help identify the preferred localization of each species and track the evolution of the interface with increasing DES concentration. The density profiles for the ChCl/EG and ChCl/cresol systems at different concentrations are presented in Figures 3 and 4.

**Figure 3:**
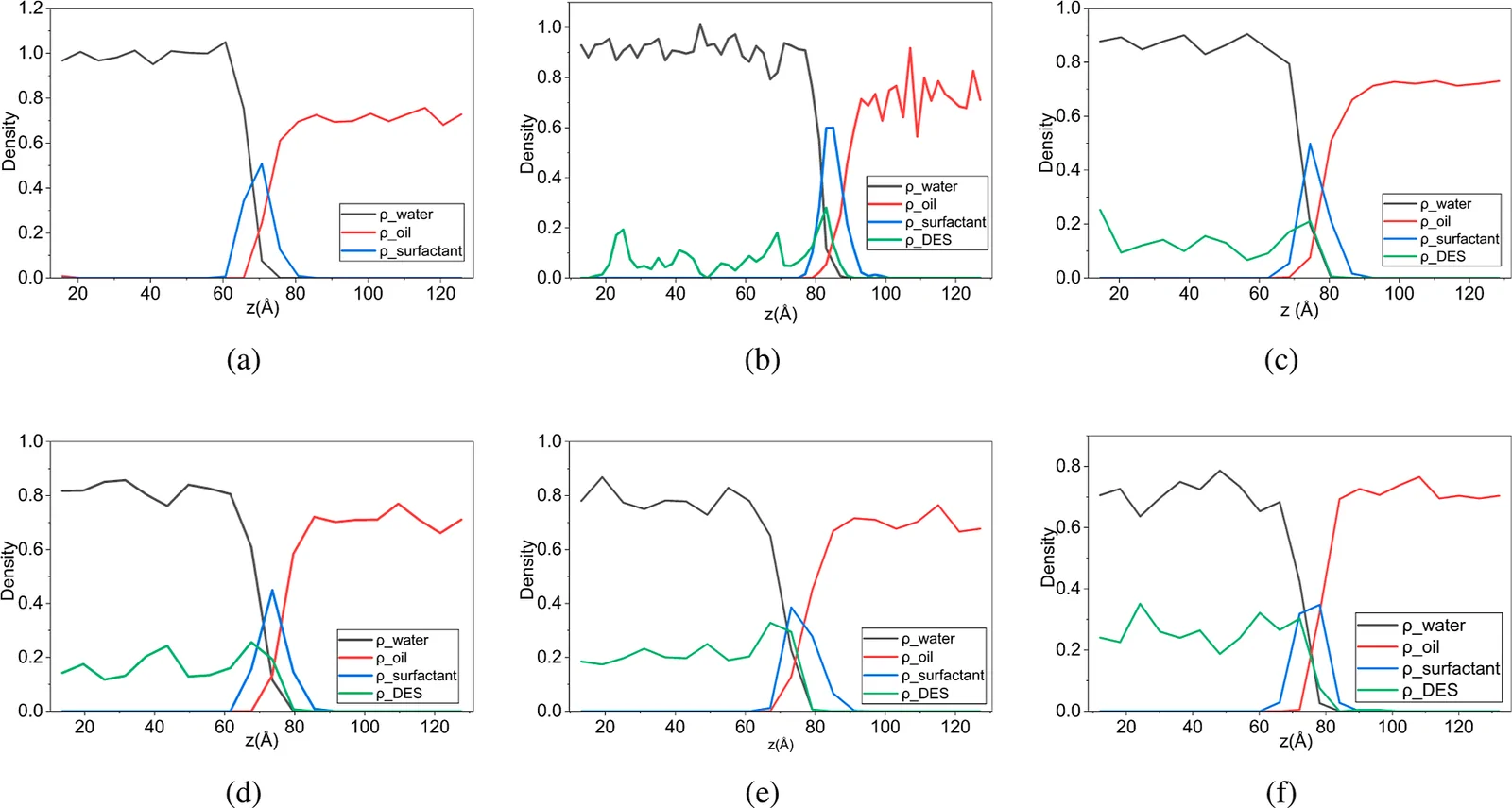
Mass density profiles along the *z*-direction for the ChCl/EG system at different DES concentrations: (a) 0%, (b) 10%, (c) 15%, (d) 20%, (e) 25%, and (f) 30%.

**Figure 4:**
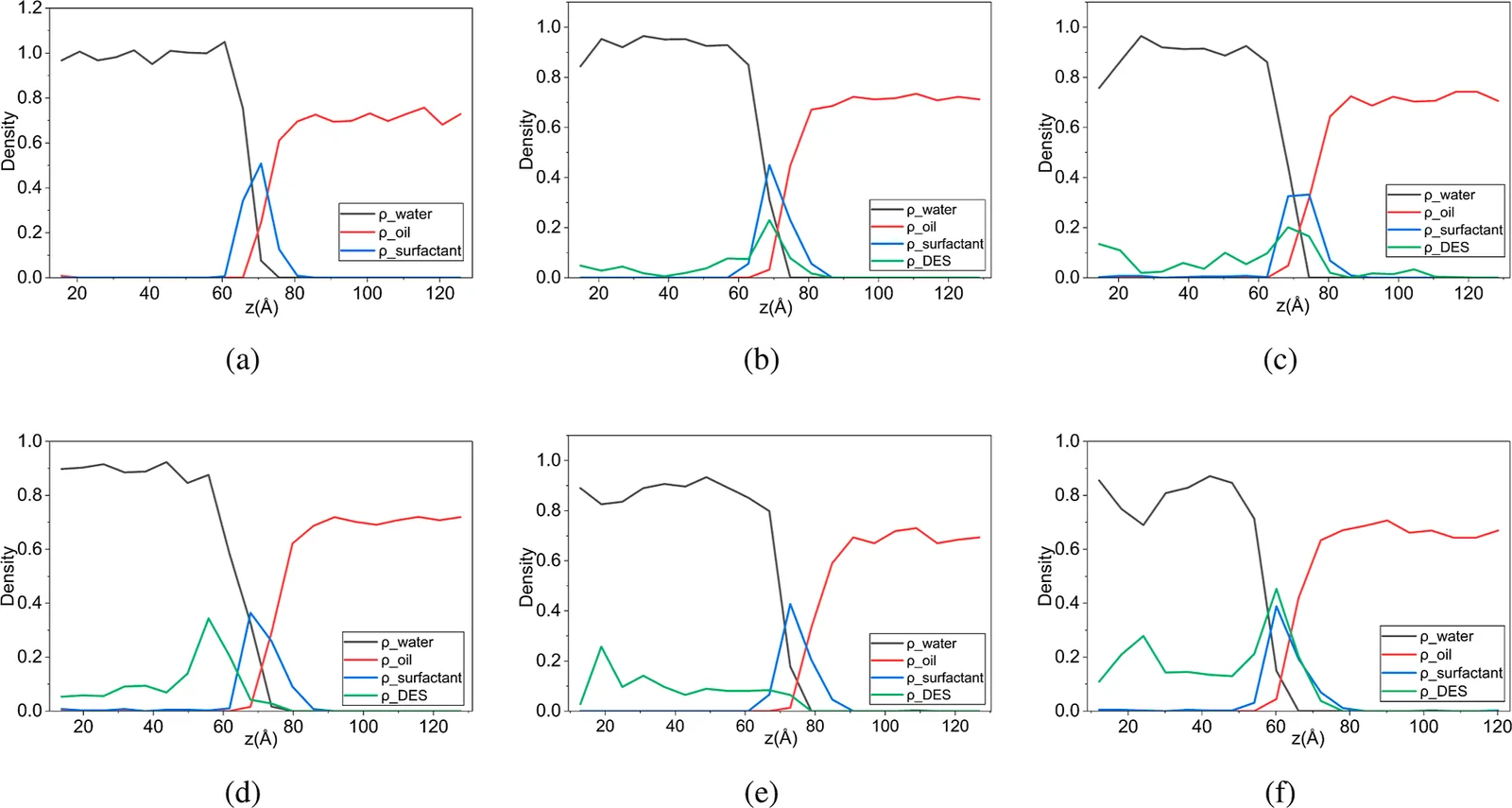
Mass density profiles along the *z*-direction for the ChCl/cresol system at different DES concentrations: (a) 0%, (b) 10%, (c) 15%, (d) 20%, (e) 25%, and (f) 30%.

In all systems, a well-defined oil–water interface is observed near the center of the simulation box. As shown in Figures 3 and 4, the water-rich and oil-rich regions maintain nearly constant bulk densities away from the interface, indicating stable phase separation and proper equilibration of the system. Near the interfacial region, the oil and water density profiles partially overlap, forming a smooth transition zone that defines the interfacial thickness.

For the ChCl/EG system (Figure 3), the overall interfacial structure remains stable as the DES concentration increases from 10% to 30%. The bulk oil-rich and water-rich regions remain largely unaffected, indicating that the addition of DES does not disrupt macroscopic phase separation. However, gradual changes are observed near the interfacial region, where the density overlap between oil and water becomes slightly broader with increasing DES concentration, suggesting enhanced interfacial structuring and redistribution of molecules near the interface. A similar behavior is observed for the ChCl/cresol system (Figure 4), where a distinct oil–water interface persists across all concentrations. However, compared to the EG-based system, the cresol-based DES exhibits relatively stronger interfacial localization, particularly at higher concentrations, which may arise from the partially amphiphilic nature of cresol and its greater affinity toward the interfacial region.

A prominent feature observed across all density profiles is the sharp surfactant density peak located near the oil–water interface. This peak reflects the amphiphilic nature of SDBS, where the hydrophilic head groups remain preferentially solvated in the aqueous phase, while the hydrophobic alkyl chains extend toward the oil-rich region.^39^ Since the surfactant concentration is maintained constant throughout all simulations, the interfacial surfactant peak serves as a useful reference for analyzing structural changes induced by increasing DES concentration. The sharp interfacial adsorption peak observed for SDBS is consistent with previous MD studies of anionic surfactants at oil–water interfaces.^11^

In contrast, the DES components exhibit concentration-dependent interfacial behavior. In both ChCl/EG and ChCl/cresol systems, the DES molecules remain predominantly confined to the aqueous phase with minimal penetration into the oil-rich region. However, as the DES concentration increases, the DES density profiles progressively shift toward the oil–water interface, indicating an enhanced interfacial accumulation on the aqueous side. This behavior, consistently observed across all systems, can be attributed to the strong hydrogen-bonding affinity of DES components toward the aqueous environment.^38^ The surfactant surface packing density was calculated as the ratio of the interfacial area to the total number of SDBS molecules adsorbed at the oil–water interface. The interfacial area, based on the simulation box dimensions of 50 × 50 Å^2^, was 2500 Å^2^. Throughout the MD simulations, all SDBS molecules remained adsorbed at the interface, resulting in a surfactant surface packing density of 167 Å^2^ molecule^–1^. Since the number of SDBS molecules was kept constant in all simulations, the surfactant surface packing density remained unchanged for both systems and at all concentrations. Furthermore, the density profiles of the ChCl/EG and ChCl/cresol systems exhibited nearly constant densities within the bulk liquid and oil phases away from the interface. No interactions between periodic interfaces were observed, indicating that finite-size effects are negligible.

#### 3.1.1 Interfacial Thickness

As the DES molecules accumulate closer to the oil–water interface, they progressively reorganize the hydrogen-bonding network within the aqueous phase. This restructuring modifies the local solvation environment surrounding the surfactant head groups, thereby stabilizing surfactant adsorption and influencing the interfacial organization. Consequently, the DES affects the interfacial structure primarily through modifications in the aqueous phase rather than through significant penetration into the oil-rich region. A comparison between the two DES systems reveals subtle but important differences in interfacial behavior. In the ChCl/EG system, the interfacial enrichment of DES components increases gradually with concentration, suggesting relatively uniform restructuring of the aqueous environment near the interface. In contrast, the ChCl/cresol system exhibits a comparatively stronger tendency for interfacial localization at higher concentrations, likely due to the partially amphiphilic nature and greater interfacial affinity of cresol molecules. These differences are also reflected in the variation of interfacial thickness values summarized in Table 3, where the cresol-based system exhibits comparatively larger fluctuations with concentration. Overall, the density profile analysis demonstrates that while the surfactant primarily governs interfacial adsorption and localization, the DES plays an important supporting role by modifying the aqueous-phase structure and influencing interfacial organization. Together, the surfactant and DES cooperatively determine the structure and stability of the ChCl-based oil–water interface.

**Table 3:** Comparison of Interfacial Thickness for ChCl/cresol and ChCl/EG Systems at Different DES Concentrations

conc (wt %)	ChCl/cresol (Å)	ChCl/EG (Å)
10	10.20	5.76
15	10.19	9.35
20	15.34	11.66
25	9.70	12.63
30	10.36	10.31

The calculated interfacial thickness values for both DES systems are summarized in Table 3. The interfacial thickness varies with DES concentration indicating concentration-dependent restructuring of the oil–water boundary in the presence of DES molecules. For the ChCl/EG system, the interfacial thickness gradually increases from 5.76 Å at 10 wt % to 12.63 Å at 25 wt % suggesting progressive broadening of the interfacial region due to enhanced hydrogen-bond-mediated organization and redistribution of water and DES molecules near the interface. Ethylene glycol strongly interacts with the aqueous phase through hydrogen bonding, leading to a more diffuse and spatially extended interfacial region. However, at 30 wt %, the interfacial thickness decreases slightly, indicating that the interface approaches a relatively saturated and structurally stabilized state at higher DES concentrations.

In comparison, the ChCl/cresol system exhibits comparatively larger fluctuations in interfacial thickness with concentration. The interfacial thickness increases significantly at 20 wt %, reaching 15.34 Å, which suggests enhanced accumulation and localization of cresol-rich domains near the oil–water interface. Owing to its partially amphiphilic nature, cresol exhibits greater affinity toward the interfacial region compared to ethylene glycol. However, at higher concentrations, the interfacial thickness decreases again, indicating the onset of stronger intermolecular association and more compact interfacial packing of cresol molecules. This behavior suggests that increasing DES concentration initially promotes interfacial broadening, whereas further concentration increase may lead to interfacial reorganization and structural compaction. Overall, the differences in interfacial thickness between the ChCl/EG and ChCl/cresol systems highlight the vital role of DES chemistry in governing interfacial organization, molecular packing, and structural heterogeneity at the oil–water interface.

### 3.2 Interfacial Properties

The interfacial tension of the surfactant–DES–water/oil interface was calculated from the microscopic pressure tensor obtained during the production stage of the simulation. The Kirkwood–Buff mechanical approach was employed because it directly relates the anisotropy of the pressure tensor across the interface to the macroscopic interfacial tension.^40^ The interfacial tension is obtained from the equation as

6 $$\gamma =\frac{L_{z}}{2}\left[P_{zz}-\frac{\left(P_{xx}+P_{yy}\right)}{2}\right]$$

where *L*_*z*_ is the box length perpendicular to the interface and *P*_*zz*_, *P*_*xx*_, and *P*_*yy*_ are the diagonal components of the pressure tensor along the *z*, *x*, and *y* directions, respectively. The factor of $\frac{1}{2}$ accounts for the presence of two identical interfaces under periodic boundary conditions. This approach provides a reliable, physics-based estimate of IFT from molecular simulation data.

The effect of DES concentration on the interfacial tension (IFT) of the surfactant–DES–water/oil system was quantified to understand the influence of DES chemistry on interfacial stabilization. The calculated IFT values for both systems at different concentrations are summarized in Table 4. For both DES systems, the IFT exhibits a clear decreasing trend with increasing DES concentration. In the absence of DES (0 wt %), the interfacial tension is approximately 12.4 mN/m, corresponding to a relatively sharp and well-defined oil–water interface. The simulated interfacial tension of the DES-free SDBS system was 12.42 mN/m. For comparison, Xu et al. reported an experimental interfacial tension of 8.03 mN/m obtained from tensiometer studies for an SDBS/n-dodecane–water system at 25 °C with an SDBS concentration of 0.007 M.^13^ The difference between the present MD simulations and the reported experimental value may arise from differences in oil composition, surfactant concentration, and other system-specific conditions. Therefore, the reported literature value is used as a benchmark rather than as a direct one-to-one validation of the present MD simulation study. Upon DES addition, a significant reduction in IFT is observed, indicating enhanced interfacial stabilization arising from cooperative surfactant–DES interactions.

**Table 4:** Interfacial Tension (IFT) as a Function of DES Concentration for DES Systems [ChCL-EG and ChCl-Cresol]^a^

concentration (wt % )	IFT (mN/m)	
	ChCl/EG	ChCl/cresol
0	12.420 ± 0.561	12.420 ± 0.561
10	10.607 ± 0.735	10.465 ± 0.242
15	10.578 ± 0.491	9.254 ± 0.489
20	9.136 ± 0.309	9.158 ± 0.201
25	9.095 ± 0.102	9.142 ± 0.223
30	8.470 ± 0.550	7.232 ± 0.337

a Uncertainties represent the standard error of the mean calculated from ten 5 ns blocks of the 50 ns MD production trajectory.

For the ChCl/EG system, the interfacial tension decreases steadily from 12.42 mN/m at 0 wt % to 8.47 mN/m at 30 wt % DES concentration. This gradual and consistent decline suggests that ethylene glycol progressively modifies the interfacial environment primarily through strengthening of the hydrogen-bonding network within the aqueous phase. As the interfacial water structure becomes increasingly organized, the surfactant head groups experience enhanced stabilization, thereby lowering the energetic cost associated with maintaining the oil–water interface. Beyond approximately 20 wt % DES concentration, the decrease in IFT becomes less pronounced, suggesting that the system approaches a saturation regime where additional DES produces only a limited interfacial effect.

A similar decreasing trend is observed for the ChCl/cresol system; however, the reduction in interfacial tension becomes more pronounced at higher DES concentrations. In this system, the IFT decreases from 12.42 mN/m at 0 wt % to 7.23 mN/m at 30 wt % DES concentration. Although cresol possesses weaker hydrogen-bonding capability than ethylene glycol,^41^ it exhibits comparatively stronger interfacial activity. This behavior can be attributed to the partially amphiphilic nature of cresol,^42^ which promotes preferential accumulation near the oil–water interface and facilitates more effective disruption of interfacial cohesion. Comparison of the two DES systems reveals distinct interfacial mechanisms governing the reduction in interfacial tension. The ChCl/EG system exhibits a smoother and more gradual decrease in IFT, consistent with the formation of a broader and relatively diffuse interfacial region, as observed in the density profile analysis. In contrast, the ChCl/cresol system attains lower IFT values at higher DES concentrations, reflecting stronger interfacial localization and enhanced interfacial association of cresol molecules. Our observations are in excellent agreement with the experimental works wherein the IFT value decreases continuously with DES (ChCl/glycerol) in 10 wt % brine solution.^43^ These observations suggest that while ethylene glycol primarily influences the interface through restructuring of the aqueous hydrogen-bonding network, cresol contributes more directly to interfacial stabilization through preferential interfacial accumulation.

To further understand the relationship between interfacial organization and interfacial tension, the interfacial energy was also analyzed for both DES systems. The interaction energy between SDBS and the DES-modified oil–water interface was calculated from the potential-energy difference as

7 $$E_{\mathrm{int}}=E_{total}-\left(E_{oil}+E_{water}+E_{SDBS}+E_{DES}\right)$$

where *E*_total_ is the potential energy of the complete system, *E*_oil+water+DES_ is the potential energy of the oil–water interface containing DES but without SDBS, and *E*_SDBS_ is the potential energy of the isolated SDBS reference system. Negative values of *E*_int_ indicate energetically favorable interaction between SDBS and the DES-modified oil–water interface. As indicated in Figure 5 in the ChCl/EG system, the interfacial energy decreases steadily with increasing DES concentration, from −43, 148.6 kJ mol^–1^ at 10 wt % to −53, 953.3 kJ mol^–1^ at 30 wt %. This gradual reduction indicates progressive stabilization of the oil–water interface with increasing EG-based DES content. The trend closely follows the corresponding decrease in IFT values, supporting the interpretation that ethylene glycol primarily modifies the interface through enhanced aqueous-phase hydrogen bonding and stabilization of surfactant head groups near the interface.

**Figure 5:**
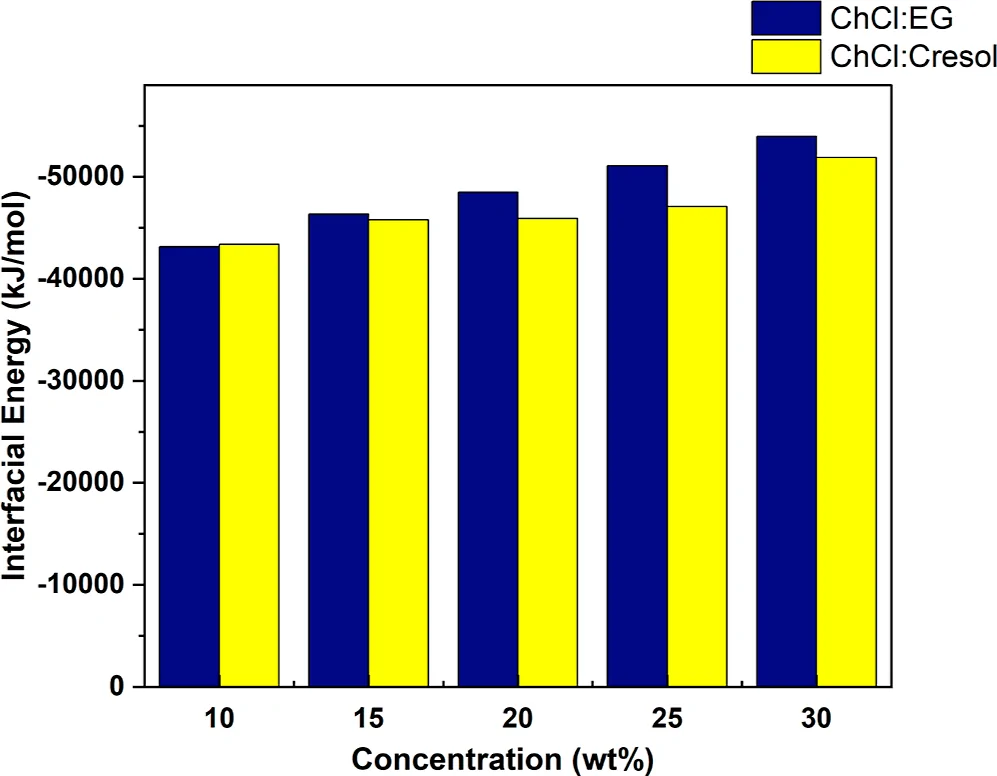
Variation of interfacial energy with DES concentration for ChCl/EG and ChCl/cresol systems.

In contrast, the ChCl/cresol system exhibits a somewhat different evolution of interfacial energy. Although the overall interfacial energy decreases with increasing DES concentration, from −43, 368.9 kJ mol^–1^ at 10 wt % to −51, 881.4 kJ mol^–1^ at 30 wt %, the variations at intermediate concentrations (15–25 wt %) are comparatively modest. This behavior suggests a concentration-dependent reorganization of the interfacial region as cresol concentration increases. Owing to its mixed hydrophilic–hydrophobic character, cresol exhibits greater interfacial affinity and a stronger tendency to localize near the oil–water interface at higher concentrations. Such enhanced interfacial association contributes to a greater reduction in interfacial energy at elevated DES loadings. Overall, the interfacial energy analysis supports the trends observed in the interfacial tension results and provides molecular-level insight into the differing interfacial behaviors of the two DES systems. In the ChCl/EG system, interfacial stabilization occurs gradually through restructuring of the aqueous hydrogen-bonding environment, leading to a relatively diffuse interfacial organization. In contrast, the ChCl/cresol system exhibits a more localized and concentration-dependent interfacial effect arising from stronger interfacial association of cresol molecules at higher DES concentrations. This distinction is consistent with the comparatively lower interfacial tension values observed for the cresol-based DES system at elevated concentrations and highlights the important role of DES chemistry in governing interfacial behavior relevant to EOR applications.

### 3.3 Hydrogen Bonding

Hydrogen bonding^44^ plays a crucial role in governing the interactions among DES components, surfactant molecules, and the oil–water interface. In this work, hydrogen bonds were identified using a geometric criterion in which the hydrogen–donor–acceptor distance was less than 3.5 Å and the donor–hydrogen–acceptor angle exceeded 150°. To obtain molecular-level insight into the interfacial behavior of the DES–surfactant systems, the average number of hydrogen bonds associated with different molecular components was analyzed as a function of DES concentration. The hydrogen-bond analysis was further used to correlate variations in intermolecular interactions with the observed trends in interfacial tension and interfacial energy.

The hydrogen bonds normalized by the number of molecules of each species present at the corresponding DES concentration (Table 1). This normalized descriptor, η_HB_, removes the confounding effect of population growth and provides a per-molecule measure of hydrogen-bonding behavior that is directly comparable across concentrations. Figure 6 presents the resulting trends for the ChCl/EG (panel a) and ChCl/cresol (panel b) systems and reveals hydrogen-bonding signatures for the two DES chemistries that are considerably more distinct than the raw counts alone suggested. In the ChCl/EG system, both ethylene glycol and choline exhibit a coupled, non-monotonic response to concentration. The normalized hydrogen-bond population of choline rises modestly from approximately 0.037 at 10 wt % to a maximum of approximately 0.043 at 20 wt %, decreases to approximately 0.040 at 25 wt %, and partially recovers to approximately 0.041 at 30 wt %. Ethylene glycol follows a closely similar trend, increasing from approximately 0.030 to 0.038 over the same 10–20 wt % window before decreasing to approximately 0.035 by 30 wt %. The parallel evolution of these two components suggests that ethylene glycol and choline draw on a shared and finite pool of hydrogen-bonding capacity within the aqueous phase: as ethylene glycol’s engagement with the hydrogen-bond network intensifies up to approximately 20 wt %, choline’s local hydrogen-bonding environment intensifies correspondingly, with both relaxing slightly at higher, more crowded concentrations. SDBS, by contrast, occupies a distinctly higher and comparatively decoupled baseline (approximately 0.068 at 10 wt %, settling to approximately 0.053–0.057 beyond 15 wt %), indicating that the hydration environment of the surfactant headgroup is only weakly perturbed by the concurrent bulk-phase reorganization of the DES components.

**Figure 6:**
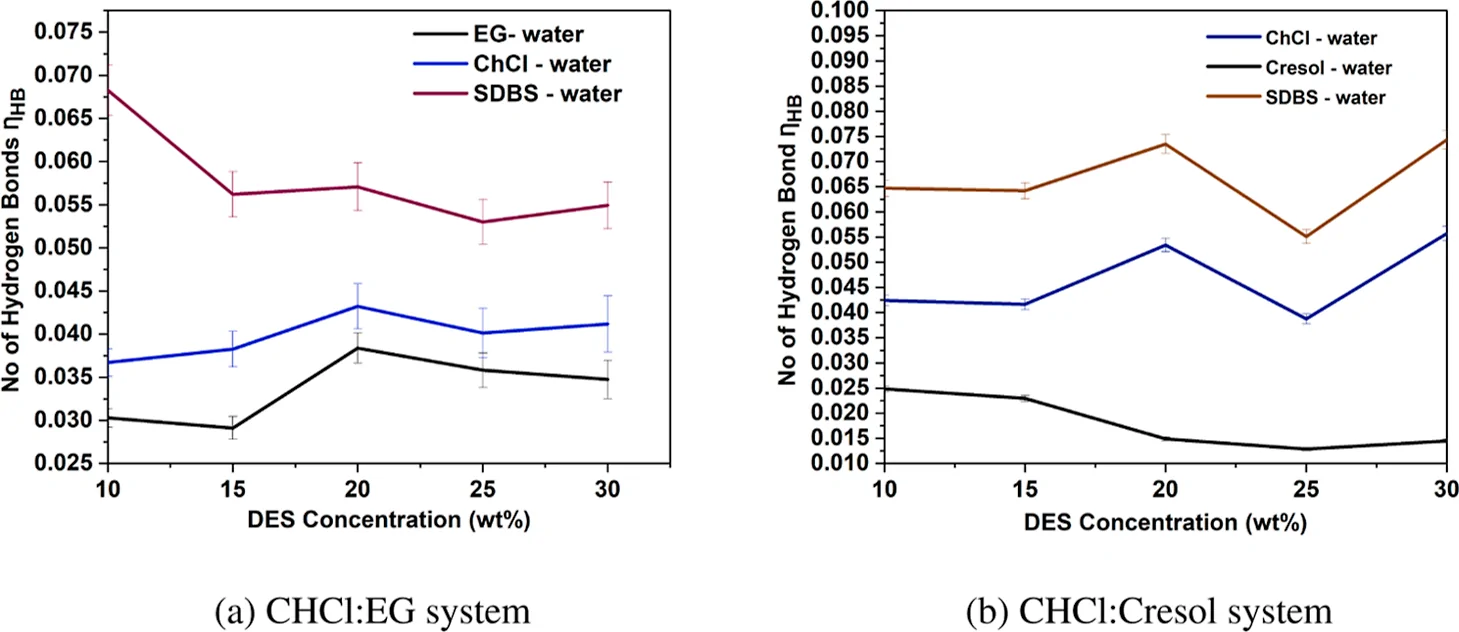
Variation in average hydrogen bonds formed by different components as a function of DES concentration for (a) ChCl/EG and (b) ChCl/cresol systems (the normalized number of hydrogen bonds was calculated by averaging over the final 10 ns of the MD production trajectory). The uncertainty represents the standard error obtained from the temporal fluctuations within this sampling interval.

The ChCl/cresol system exhibits a markedly different normalized hydrogen-bonding behavior. Choline again shows a rising-then-fluctuating trend, increasing from approximately 0.042 at 10 wt % to approximately 0.053 at 20 wt %, decreasing to approximately 0.039 at 25 wt %, and subsequently increasing to approximately 0.056 at 30 wt %, broadly mirroring its behavior in the EG-based system. Cresol, however, deviates sharply from this pattern: its normalized hydrogen-bond population decreases steadily and substantially, from approximately 0.025 at 10 wt % to a minimum of approximately 0.013 at 25 wt %, followed by a marginal increase at 30 wt %. This trend is opposite that of ethylene glycol and constitutes the most distinctive feature of the normalized analysis. A decreasing per-molecule hydrogen-bond count with increasing concentration indicates that a growing fraction of cresol molecules, on average, spends less time in a hydrogen-bonded state with their surroundings, consistent with the partially amphiphilic character of cresol, which may allow a portion of the population to reside in environments comparatively poor in hydrogen-bond partners.

The normalized hydrogen-bond data indicate that the bulk aqueous-phase hydrogen-bonding engagement of cresol weakens with increasing DES concentration; this is a direct and well-supported reading of Figure 6. The declining η_HB_ for cresol is consistent with, and lends additional support to, the interfacial-accumulation picture developed from the density-profile, radial distribution function (RDF), and coordination-number analyses.

SDBS in the ChCl/cresol system exhibits the most pronounced fluctuations of any species examined (approximately 0.065 at 10 wt %, rising to 0.073 at 20 wt %, decreasing to 0.055 at 25 wt %, and increasing again to 0.074 at 30 wt %), broadly tracking the oscillatory behavior of choline. This co-variation between the surfactant headgroup and choline, which is absent in the corresponding EG-based system, indicates a more actively reorganizing local hydrogen-bond network in the cresol-based system—one in which the hydration state of the surfactant headgroup is more tightly coupled to the concentration-dependent restructuring of the DES than is observed for ethylene glycol.

The ChCl/EG system behaves as a comparatively cohesive unit, in which ethylene glycol and choline strengthen and relax their hydrogen bonding in tandem while SDBS remains comparatively unperturbed. The ChCl/cresol system, in contrast, shows cresol progressively disengaging from the bulk hydrogen-bond network even as choline and SDBS undergo more pronounced, coupled fluctuations.

The temporal persistence of the hydrogen-bond network was quantified using the hydrogen-bond autocorrelation function, *C*(*t*), which measures the probability that a hydrogen bond present at an initial time remains correlated at a later time. The autocorrelation function was calculated as

8 $$C\left(t\right)=\frac{\left\langle h\left(0\right)h\left(t\right)\right\rangle }{\left\langle h{\left(0\right)}^{2}\right\rangle }$$

where *h*(*t*) is a binary hydrogen-bond indicator function, with *h*(*t*) = 1 when a hydrogen bond exists between the selected donor–acceptor pair at time *t* and *h*(*t*) = 0 otherwise, and ⟨·⟩ denotes an ensemble average over all hydrogen bonds and time origins. Thus, *C*(0) = 1, and the decay of *C*(*t*) describes the loss of temporal correlation of the hydrogen-bond network. The characteristic hydrogen-bond relaxation time, τ, was obtained by integrating the autocorrelation function over time

9 $$\tau =\int_{0}^{\infty }C\left(t\right)dt$$

where τ represents the relaxation time over which the hydrogen-bond correlations persist. A larger τ therefore indicates a more slowly relaxing and dynamically persistent hydrogen-bond environment. The h-bond autocorrelation function for different h-bonded pairs such as ChCl-water, cresol-water, SDBS-water, and EG-water for both the DES systems is shown in Figure S2 of the Supporting Information section.

The hydrogen-bond autocorrelation analysis provides further insight into the temporal persistence of intermolecular interactions within the DES–surfactant systems. As shown in Table 5, the characteristic hydrogen-bond relaxation time, τ obtained from the autocorrelation function, shows an increase with increasing DES concentration from 10 to 30 wt %. In the ChCl/cresol system, the τ values of cresol-water, ChCl-water, and SDBS-water h-bonded pairs increase from 0.6116 to 0.6448, 0.8980 to 1.1381, and 1.1607 to 1.2272, respectively. The relatively larger increase observed for ChCl-water indicates that the hydrogen-bond correlations involving the choline environment become more persistent at higher DES concentration. In comparison, the changes in the relaxation times of cresol and SDBS are comparatively smaller.

**Table 5:** Hydrogen-Bond Relaxation Time (τ) for the Different Molecular Components with Water at 10 and 30 wt % DES Concentration

relaxation time (τ) (ps) ChCl/cresol			relaxation time (τ) (ps) ChCl/EG		
H-bonded pair	10 wt %	30 wt %	H-bonded pair	10 wt %	30 wt %
cresol-water	0.6116	0.6448	ethylene glycol-water	0.5580	0.5836
ChCl-water	0.8980	1.1381	ChCl-water	0.9342	1.2751
SDBS-water	1.1607	1.2272	SDBS-water	1.2324	1.6346

A similar trend is observed for the ChCl/EG system. The relaxation time of ethylene glycol-water h-bonded pair increases slightly from 0.5580 to 0.5836 ps, whereas ChCl-water exhibits a more pronounced increase from 0.9342 to 1.2751 ps. Notably, SDBS-water pair exhibits the largest relaxation time in this system, increasing from 1.2324 ps at 10 wt % to 1.6346 ps at 30 wt %. The relatively large τ values of SDBS-water indicate that hydrogen-bond correlations persist for longer durations at higher DES concentrations, suggesting a more dynamically persistent interfacial hydrogen-bond environment.

It is important to distinguish between the hydrogen bond population and its temporal persistence. Although the average number of hydrogen bonds involving SDBS changes only moderately with the DES concentration, the corresponding autocorrelation time increases, particularly in the ChCl/EG system. Thus, the concentration-dependent interfacial behavior cannot be attributed solely to changes in the number of hydrogen bonds; rather, the lifetime and persistence of the existing hydrogen-bond network may also contribute to the molecular organization of the interface. The longer-lived hydrogen bond correlations observed at higher DES concentrations are therefore consistent with the formation of a more dynamically persistent interfacial environment.

### 3.4 Intermolecular Structure and Pair Distribution Functions

The RDF, *g*(*r*), was calculated from the MD trajectories to determine the probability of finding a neighboring atom at a distance *r* from a reference atom relative to an ideal gas distribution.^45^ The RDFs were obtained by averaging the pairwise distance distributions over the equilibrated portion of the simulation trajectories. The coordination number (CN)^46^ was evaluated by integrating the RDF up to the first minimum following the primary peak according to

10 $$N=4\pi \rho \int_{0}^{r_{\min}}g\left(r\right)r^{2}dr$$

where ρ represents the number density of the coordinating species. The CN obtained from pair distribution function for different interatomic pairs is displayed in Table 6.

**Table 6:** Coordination Numbers for DES–Water Interactions at Different DES Concentrations

DES conc.	SDBS head–water	SDBS head–water	EG(O)–H_*w*_	EG(O)–O_*w*_	cresol(O)–H_*w*_	cresol(O)–O_*w*_
(wt %)	ChCl/cresol	ChCl/EG	ChCl/EG	ChCl/EG	ChCl/cresol	ChCl/cresol
10	1.8709	1.0048	0.9044	2.5697	0.6954	2.1717
15	2.6872	0.9575	0.8679	2.4810	0.6337	2.0864
20	2.6677	0.9247	0.8427	2.4162	0.5577	1.8522
25	1.7170	0.8832	0.8025	2.3041	0.5019	1.7465
30	2.5918	0.8612	0.7616	2.1932	0.5736	1.9562

The RDFs between the SDBS headgroup and water molecules shown in Figure 7 exhibit a distinct first hydration shell at approximately 1.65 Å for both DES systems, indicating strong hydrogen-bond interactions between the surfactant headgroup and surrounding water molecules. The ChCl: Cresol system shows a sharper first peak followed by a deeper minimum, indicating that water molecules are more narrowly distributed within the first coordination shell around the SDBS headgroup. This narrower shell is consistent with a more localized local packing of water around the headgroup. In contrast, the broader peak and slower decay observed for ChCl/EG indicate a wider coordination shell, with water distributed over a broader range of distances. This behavior suggests that ethylene glycol participates more extensively in the surrounding hydrogen-bonding network, thereby modifying direct headgroup–water interactions. Furthermore, the more pronounced long-range structural correlations observed in the cresol-based DES system indicate enhanced water organization near the surfactant interface compared to the EG-based system. In the ChCl/EG system, the CN of the SDBS headgroup–water pair decreases progressively with increasing DES concentration, indicating a reduction in the number of water molecules within the first coordination shell. This trend is consistent with the progressive redistribution of DES components toward the interfacial aqueous region, which modifies the local distribution of water around the SDBS headgroup. In contrast, the ChCl/cresol system exhibits a non-monotonic CN variation (Table 6), reflecting concentration-dependent reorganization of the interfacial hydration environment. In the ChCl/EG system, the CN of the SDBS headgroup–water pair decreases progressively with increasing DES concentration (Table 6), indicating a reduction in the number of water molecules within the first coordination shell. This trend is consistent with the progressive redistribution of DES components toward the interfacial aqueous region, which modifies the local distribution of water around the SDBS headgroup. In contrast, the ChCl/cresol system exhibits a non-monotonic CN variation (Table 6), reflecting concentration-dependent reorganization of the interfacial hydration environment.

**Figure 7:**
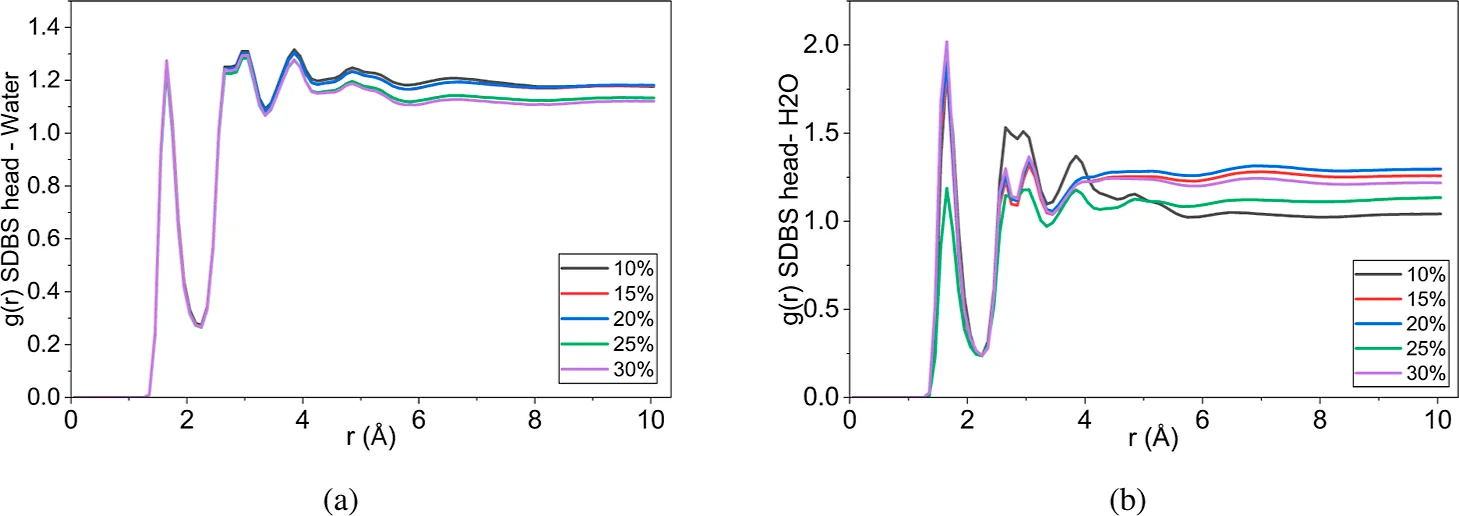
Pair distribution function corresponding to different pairs (a) SDBS (head)-water for ChCl/EG and (b) SDBS (head)-water for ChCl/cresol.

The first peak of the EG oxygen–water hydrogen (Figure 8a) RDF appears 1.85 Å, indicating preferential localization of water hydrogen atoms around the ethylene glycol oxygens. The distinct first-shell feature reflects a relatively localized spatial arrangement of water around the hydroxyl oxygen. The pronounced first peak suggests a well-defined local hydration structure surrounding ethylene glycol molecules, with water molecules preferentially arranged near the hydroxyl groups. This behavior reflects the strong interaction of ethylene glycol with the aqueous hydrogen-bonding network and highlights its role as an effective hydrogen-bond acceptor within the aqueous phase. The CN for the EG­(Oxy)-HW pair shows a small decrease with DES concentration and this behavior may be attributed to EG–EG association by hydrogen-bonding. The EG­(Oxy)–OW RDF shown in Figure 8b exhibits a first coordination peak at 2.85 Å, indicating a more spatially distributed coordination environment compared with the more localized EG­(Oxy)–HW first shell. With increasing DES concentration, the gradual reduction in peak intensity accompanied by a smoother decay indicates a broadening and redistribution of the EG–water spatial correlations rather than simply a reduction in interaction strength. These changes demonstrate the reorganization of the local EG–water environment rather than providing insights for a change in the strength of EG–water interactions. Moreover, the shorter characteristic separation for the EG­(Oxy)–HW compared to EG­(Oxy)-OW pair indicates that water hydrogen atoms approach the ethylene glycol oxygen more closely than water oxygen atoms. The coordination numbers of both EG­(Oxy)–HW and EG­(Oxy)–OW decrease progressively with increasing DES concentration, demonstrating a reduction in the integrated population of water within the first coordination shell of ethylene glycol. The simultaneous decrease in CN for both EG­(Oxy)–HW and EG­(Oxy)–OW indicates that the local water coordination around EG becomes progressively less populated as the DES concentration increases.

**Figure 8:**
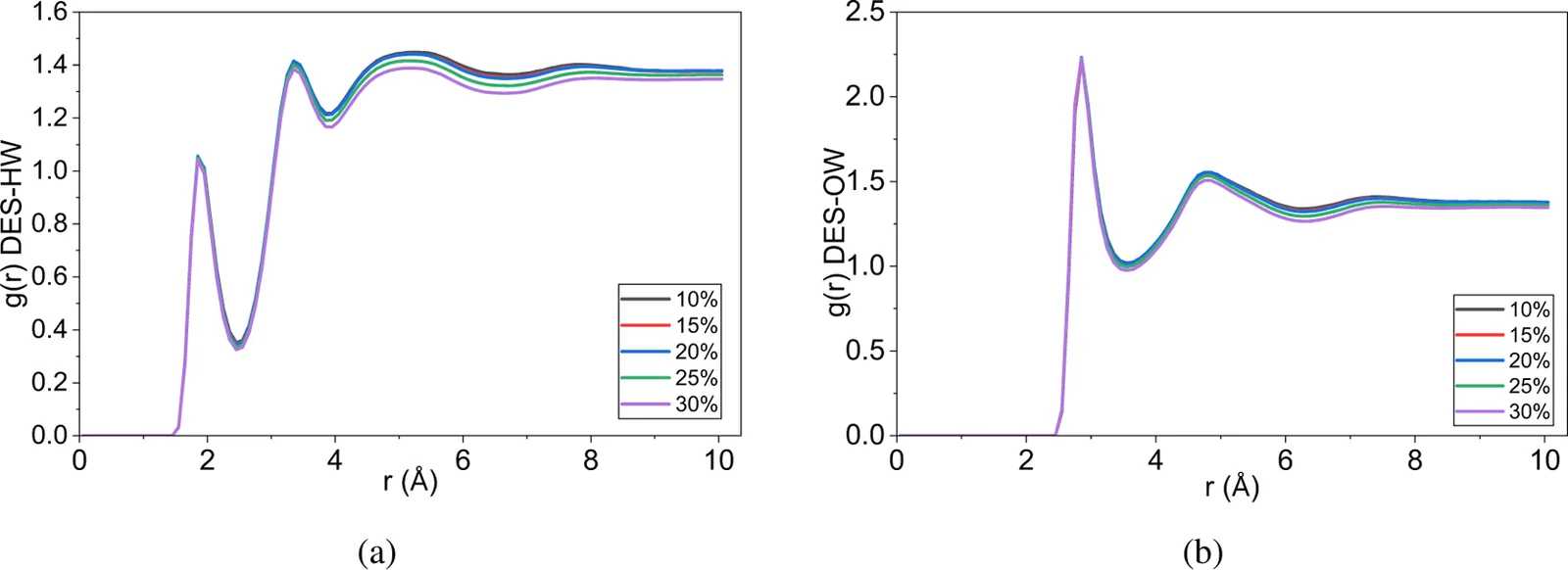
Pair distribution between ethylene glycol and water (a) EG­(xy)-HW and (b) EG­(Oxy)-OW.

The pair distribution function for the pair ChCl/cresol shown in Figure 9 provides insight into the interactions between cresol molecules and surrounding water molecules. The RDF corresponding to the interaction between the hydroxyl oxygen of cresol and the hydrogen atoms of water (Figure 9a) exhibits a distinct first peak at approximately 1.95 Å, indicating strong hydrogen-bond interactions between cresol and water molecules. The pronounced first peak suggests a well-defined local hydration structure surrounding cresol molecules, reflecting their effective participation in the aqueous hydrogen-bonding network. Such interactions contribute to localized structural organization near the oil–water interface. As the DES concentration increases, the peak intensity in both RDF profiles (Figure 9b) becomes more pronounced and the structural correlations extend to larger distances. The coordination numbers for both cresol­(O)–HW and cresol­(O)–OW interactions decrease from 10–25 wt %, indicating progressive depletion of water molecules from the first hydration shell of cresol due to increased interfacial accumulation and cresol–cresol association. At 30 wt %, a slight increase in CN is observed, suggesting interfacial reorganization and partial rehydration of cresol hydroxyl groups through the formation of localized water–cresol hydrogen-bond networks.

**Figure 9:**
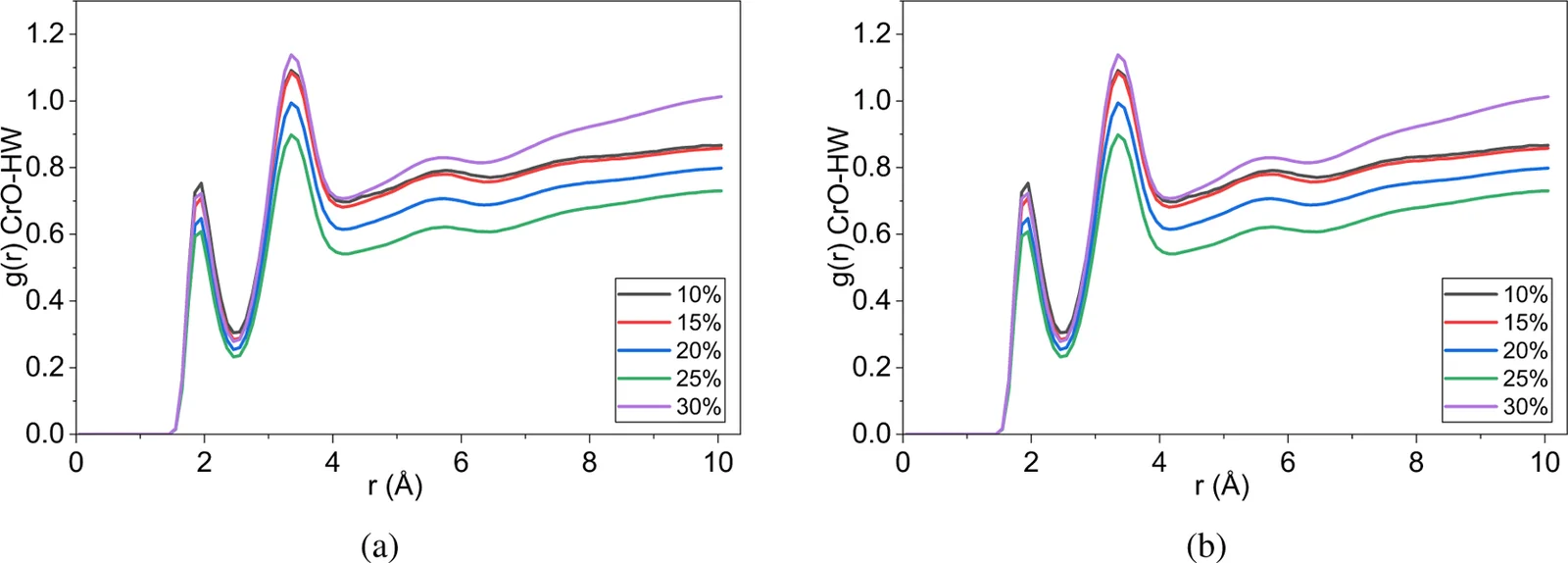
Pair distribution between cresol and water (a) Cr­(Oxy)-HW and (b) Cr­(Oxy)-OW.

The RDFs for the SDBS headgroup oxygen-water hydrogen pair, $g_{SDBS,O-H_{w}}\left(r\right)$ , are shown for different DES concentrations. A pronounced first peak is observed at approximately 1.6–1.7 Å, indicating a strong and well-defined local interaction between the SDBS headgroup and water molecules. The subsequent minimum near 2.2–2.4 Å separates the first coordination shell from the more distant solvation environment. A second peak around 3.0–3.2 Å indicates additional structural ordering in the surrounding hydration layer. For Figure 10a, the RDF profiles exhibit relatively small variations with DES concentration, suggesting that the local SDBS–water structure remains comparatively stable. In contrast, Figure 10b shows a stronger concentration dependence, particularly in the intensity of the first and second peaks, indicating greater reorganization of the interfacial hydration environment with increasing DES concentration. Overall, the RDFs confirm that DES concentration influences the local ordering and hydration structure around the SDBS headgroup.

**Figure 10:**
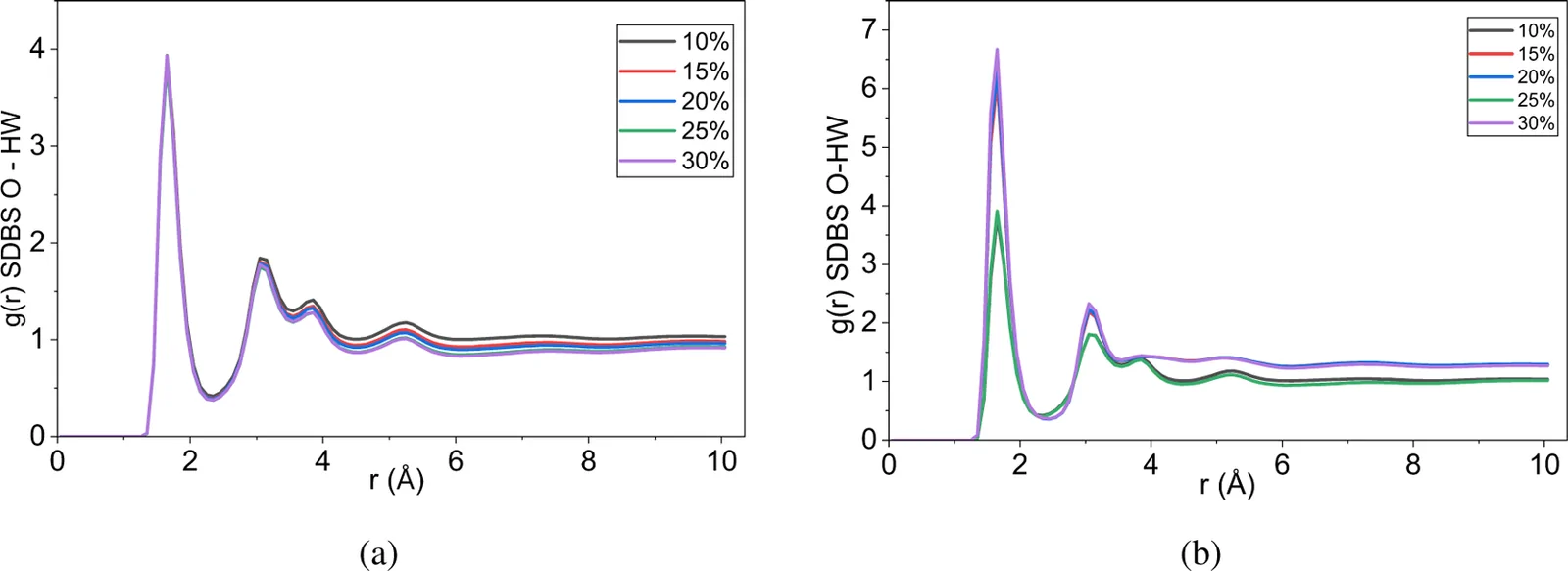
Pair distribution function corresponding to different pairs: (a) SDBS­(O)–water HW for ChCl/EG; (b) SDBS­(O)–water HW for ChCl/cresol.

### 4.1 Excess Entropy

The two-body excess entropy derived from the RDF^47^ was used to quantify the degree of local structural ordering induced by choline chloride (ChCl)-based DESs in the aqueous phase. The two-body local excess entropy, *S*_ex_, was estimated from the pair correlation function as^48^

11 $$S_{ex}\approx -2\pi \rho \int_{0}^{\infty }\left[g\left(r\right)\ln g\left(r\right)-g\left(r\right)+1\right]r^{2}dr$$

where ρ is the number density and *g*(*r*) is the RDF. In practice, *g*(*r*) is obtained from MD simulations, and the integral is evaluated numerically over the radial distance. This formulation relates pairwise intermolecular correlations to the degree of structural ordering relative to an ideal gas reference state. More negative values of the two-body excess entropy correspond to stronger intermolecular ordering and reduced configurational freedom within the aqueous phase.

In the present work, the RDF-derived two-body excess entropy was employed primarily as a comparative structural ordering descriptor to examine how DES molecules influence intermolecular organization within the interfacial aqueous environment. Owing to the heterogeneous and interfacial nature of the system, complete convergence of *g*(*r*) to unity at large distances is not observed. Therefore, the calculated excess entropy values are interpreted qualitatively to compare relative changes in structural ordering across different DES concentrations rather than as rigorous thermodynamic entropy values. For the ChCl/EG–water system, the RDF-derived two-body excess entropy values remain strongly negative across all concentrations, indicating enhanced intermolecular ordering within the aqueous phase. The magnitude of the excess entropy increases progressively from 10 to 20 wt %, reaching its most negative value at 20 wt %, which suggests enhanced hydrogen-bond-mediated organization within the aqueous environment. This behavior is consistent with the strong hydrogen-bonding capability of ethylene glycol, which participates extensively in the surrounding hydrogen-bond network through interactions with water molecules and chloride ions. At higher concentrations (25–30 wt %), the slight reduction in excess entropy magnitude suggests partial disruption of this relatively ordered arrangement, likely associated with increased DES–DES association and local packing effects at elevated concentrations.

The RDF-derived two-body excess entropy values for both DES systems are summarized in Table 7. The choline headgroup–water interactions in the ChCl/EG system exhibit comparatively smaller negative excess entropy values that vary gradually with increasing DES concentration. This behavior indicates that although the choline headgroups remain hydrated, their contribution to local aqueous ordering is comparatively weaker than that of ethylene glycol. Overall, the ChCl/EG system promotes relatively uniform and cooperative intermolecular organization within the aqueous phase, primarily governed by hydrogen-bond interactions.

**Table 7:** Excess Entropy Values for ChCl/EG and ChCl/Cresol Systems at Different DES Concentrations

concentration (% wt)	ChCl/cresol		ChCl/EG	
	DES–water	SDBS head–water	DES–water	SDBS head–water
	(JK^–1^ mol^–1^)	(JK^–1^ mol^–1^)	(JK^–1^ mol^–1^)	(JK^–1^ mol^–1^
10	–1036.60	–507.86	–200.49	–261.85
15	–799.25	–143.10	–228.56	–240.46
20	–833.70	–177.98	–251.33	–244.57
25	–981.41	–72.89	–257.18	–203.46
30	–618.92	–177.99	–255.66	–212.07

A contrasting behavior is observed for the ChCl/cresol–water system. At lower concentrations, the DES–water excess entropy values are comparatively less negative, indicating weaker intermolecular ordering within the aqueous phase. This behavior may be associated with the bulkier and partially hydrophobic nature of cresol, which limits the formation of extended hydrogen-bond-mediated organization with surrounding water molecules and results in comparatively greater structural flexibility within the aqueous environment. Interestingly, the SDBS headgroup–water excess entropy values in the ChCl/cresol system exhibit a pronounced decrease at intermediate concentrations (15–20 wt %), suggesting enhanced localized ordering of water molecules near the ionic headgroups. This behavior indicates that as cresol perturbs the surrounding hydrogen-bonding environment, hydration becomes increasingly localized around polar and ionic sites. At higher concentrations (25–30 wt %), the excess entropy values for both DES–water and headgroup–water interactions become more negative again, indicating further reorganization of the local hydration environment arising from increased intermolecular association of cresol molecules within the aqueous phase.

Overall, these results demonstrate contrasting intermolecular ordering behaviors between the two DES systems. The ChCl/EG system promotes relatively uniform and cooperative aqueous-phase organization primarily through hydrogen-bond interactions. In contrast, the ChCl/cresol system exhibits a more heterogeneous and concentration-dependent hydration environment characterized by localized ordering near ionic regions and comparatively disrupted bulk aqueous organization. These observations further highlight the important role of hydrogen-bond donor chemistry in governing intermolecular organization and interfacial behavior in DES–water systems.

## 5 Conclusions

This study employs MD simulations to investigate how DESs work in combination with an anionic surfactant to modify the oil–water interface relevant to c-EOR. By comparing two DES systems over a range of concentrations, we clearly demonstrate how DES chemistry governs interfacial structure and behavior.

Across all systems, a stable oil–water interface is observed, with the surfactant consistently anchoring itself at the interface due to its amphiphilic nature. The DES components remain largely confined to the aqueous phase; however, as their concentration increases, they progressively migrate toward the interface on the water side. Rather than directly entering the oil phase, the DESs influence interfacial properties by restructuring the surrounding water environment. This indirect effect plays a crucial role in stabilizing the surfactant and modifying interfacial characteristics.

Both DES systems lead to a clear and systematic reduction in interfacial tension, confirming a strong synergistic interaction between the surfactant and DES. The ethylene glycol-based DES exhibits a gradual decrease in interfacial tension, reflecting a uniform strengthening of hydrogen bonding within the aqueous phase. In contrast, the cresol-based DES produces a more pronounced reduction at higher concentrations. This behavior can be attributed to the partially amphiphilic nature of cresol, which allows it to accumulate closer to the oil–water interface and participate more directly in interfacial stabilization. The confidence in the absolute quantitative IFT values is limited by the absence of direct experimental validation of the corresponding DES–surfactant–oil–water systems, whereas the observed concentration-dependent trends and the comparative differences between the two DES chemistries provide the primary molecular-level conclusions of this study. We further acknowledge that experimental measurements of the corresponding SDBS–DES–oil–water systems would be valuable for quantitatively validating the predicted IFT values and assessing their broader generalizability.

Hydrogen-bonding and structural analyses provide a molecular-level explanation of these trends. Ethylene glycol primarily enhances bulk water structuring, thereby indirectly supporting the surfactant headgroups at the interface. Cresol, on the other hand, contributes through both hydrogen bonding and direct interfacial interactions, leading to more localized and concentration-dependent effects. Excess entropy analysis further corroborates these findings, indicating that ethylene glycol promotes a more uniform and cooperative arrangement of water molecules, while the cresol-based system generates a more heterogeneous hydration environment concentrated near the interface.

Overall, these results demonstrate that DESs are not merely passive additives but active components that significantly influence interfacial behavior. By carefully selecting the HBD, the interactions between DESs, water, and the oil–water interface can be deliberately tuned, enabling more efficient interfacial tension reduction and potentially lowering surfactant requirements. These molecular-level insights provide a strong foundation for the rational design of more effective and sustainable DES–surfactant formulations for EOR applications.

## Supplementary Material



## Notes

Complete LAMMPS force field parameters including Lennard–Jones, bond, angle, and dihedral coefficients for choline chloride/cresol (ChCl/cresol) and choline chloride/ethylene glycol (ChCl/EG) systems; convergence plot of system density and potential energy; and hydrogen-bond autocorrelation (PDF)

## References

[1] International Energy Agency . World energy balances 2024 – overview. https://www.iea.org/reports/world-energy-balances-overview/world, 2024; (accessed Nov 08, 2025).

[2] Prajapati P., Guo R., Cai A., Prashad R. (2026). Navigating the energy transition
in india: challenges and opportunities towards sustainable energy
goal. Water-Energy Nexus.

[3] Olabi A. G., Elsaid K., Obaideen K., Abdelkareem M. A., Rezk H., Wilberforce T., Maghrabie H. M., Sayed E. T. (2023). Comparisons, challenges and barriers, sustainability
indicators, and the contribution to un sustainable development goals. Int. J. Thermofluids.

[4] Muradov N. (2017). Low to near-zero
co2 production of hydrogen from fossil fuels: Status and perspectives. Int. J. Hydrogen Energy.

[5] Şahin S. (2019). Tailor-designed
deep eutectic liquids as a sustainable extraction media: An alternative
to ionic liquids. J. Pharm. Biomed. Anal..

[6] Gbadamosi A. O., Junin R., Manan M. A., Agi A., Yusuff A. S. (2019). An overview
of chemical enhanced oil recovery: recent advances and prospects. Int. Nano Lett..

[7] Green, D. W. ; Paul Willhite, G. Enhanced Oil Recovery; Society of Petroleum Engineers: Richardson, TX, 1998.

[8] L. W., Lake . Enhanced Oil Recovery, 2 ed.; Society of Petroleum Engineers: Richardson, TX, 2014.

[9] Green, D. W. ; Paul Willhite, G. Enhanced Oil Recovery Processes, 2 ed.; Society of Petroleum Engineers: Richardson, Texas, 2018.

[10] Pu W., He Y., Wu T., He W., Chen Q., Yang F., Jiang H., Hou S. (2026). Emulsification and interfacial characteristics
of different surfactants enhances heavy oil recovery: experimental
evaluation and molecular dynamics simulation study. J. Dispersion Sci. Technol..

[11] Chen Z., Zhu Y., Zhong J., Liu P., Wang Y., Yu H., Zhang L., Ma L., Sun D., Xia K. (2025). Molecular
dynamics simulation of the effects of anionic–nonionic surfactants
on interfacial properties of the oil–water interface. ACS Omega.

[12] Gong L., Liao G., Luan H., Chen Q., Nie X., Liu D., Feng Y. (2020). Oil solubilization in sodium dodecylbenzenesulfonate
micelles: New insights into surfactant enhanced oil recovery. J. Colloid Interface Sci..

[13] Xu J., Zhang Y., Chen H., Wang P., Xie Z., Yao Y., Yan Y., Zhang J. (2013). Effect of surfactant headgroups on
the oil/water interface: An interfacial tension measurement and simulation
study. J. Mol. Struct..

[14] Sheng, J. J. Modern Chemical Enhanced Oil Recovery; Gulf Professional Publishing: Oxford, 2015.

[15] Olajire A. A. (2014). Review
of asp eor (alkaline surfactant polymer enhanced oil recovery) technology
in the petroleum industry: Prospects and challenges. Energy.

[16] Abdurrahman A., Shuwa S. M., Dabai F. N., Oyegoke T., Igbafe A. I., Orodu O. D., Jibril B. Y. (2025). Elucidating
recovery and mechanism
of deep eutectic solvents for heavy crude oil via experimental and
computational analysis. J. Mol. Liq..

[17] Smith E. L., Abbott A. P., Ryder K. S. (2014). Deep eutectic solvents
(dess) and
their applications. Chem. Rev..

[18] Abbott A. P., Capper G., Davies D. L., Rasheed R. K. (2003). Novel solvent properties
of choline chloride/urea mixtures. Chem. Commun..

[19] El-Hamarnah Y., Al-Salti N., Al-Wahaibi Y. (2020). Interfacial
tension reduction using
deep eutectic solvents for enhanced oil recovery. J. Mol. Liq..

[20] Al-Shakhs M., Al-Maamari R. (2022). Experimental
investigation of deep eutectic solvents
as eor additives. Energy Fuels.

[21] Zhou Y., Zhang L., Wang H. (2021). Molecular
dynamics study of deep
eutectic solvents at oil–water interfaces. J. Mol. Liq..

[22] Rahimi A., Shokrollahi A. (2022). Molecular
insight into wettability alteration by deep
eutectic solvents. Fuel.

[23] Ahmed S., Al-Wahaibi Y., Ayatollahi S. (2023). Hybrid surfactant–deep eutectic
solvent systems for enhanced oil recovery. Energy
Reports.

[24] Badwaik P., Chobe S., Malani A. (2023). Investigation
of light oil–rock
interactions using molecular dynamics simulations. Energy Fuels.

[25] Mark P., Nilsson L. (2001). Structure and dynamics
of the tip3p, spc, and spc/e
water models at 298 k. J. Phys. Chem. A.

[26] Jasiok B., Pribylov A. A., Postnikov E. B., Friant-Michel P., Millot C. (2024). Thermophysical properties of the
spc/e model of water
between 250 and 400 k at pressures up to 1000 mpa. Fluid Phase Equilib..

[27] Chen Z., Zhu Y., Zhong J., Liu P., Wang Y., Yu H., Zhang L., Ma L., Sun D., Xia K. (2025). Molecular
dynamics simulation of the effects of anionic–nonionic surfactants
on interfacial properties of the oil–water interface. ACS Omega.

[28] Frisch, M. J. ; Trucks, G. W. ; Schlegel, H. B. ; Scuseria, G. E. ; Robb, M. A. ; Cheeseman, J. R. ; Scalmani, G. ; Barone, V. ; Petersson, G. A. ; Nakatsuji, H. ; Li, X. ; Caricato, M. ; Marenich, A. V. ; Bloino, J. ; Janesko, B. G. ; Gomperts, R. ; Mennucci, B. ; Hratchian, H. P. ; Ortiz, J. V. ; Izmaylov, A. F. ; Sonnenberg, J. L. ; Williams-Young, D. ; Ding, F. ; Lipparini, F. ; Egidi, F. ; Goings, J. ; Peng, B. ; Petrone, A. ; Henderson, T. ; Ranasinghe, D. ; Zakrzewski, V. G. ; Gao, J. ; Rega, N. ; Zheng, G. ; Liang, W. ; Hada, M. ; Ehara, M. ; Toyota, K. ; Fukuda, R. ; Hasegawa, J. ; Ishida, M. ; Nakajima, T. ; Honda, Y. ; Kitao, O. ; Nakai, H. ; Vreven, T. ; Throssell, K. ; Montgomery, J. A., Jr. ; Peralta, J. E. ; Ogliaro, F. ; Bearpark, M. J. ; Heyd, J. J. ; Brothers, E. N. ; Kudin, K. N. ; Staroverov, V. N. ; Keith, T. A. ; Kobayashi, R. ; Normand, J. ; Raghavachari, K. ; Rendell, A. P. ; Burant, J. C. ; Iyengar, S. S. ; Tomasi, J. ; Cossi, M. ; Millam, J. M. ; Klene, M. ; Adamo, C. ; Cammi, R. ; Ochterski, J. W. ; Martin, R. L. ; Morokuma, K. ; Farkas, O. ; Foresman, J. B. ; Fox, D. J. Gaussian 16 Revision C.01; Gaussian Inc.: Wallingford CT, 2016.

[29] Roy, D. ; Keith, T. A. ; Millam, J. M. Gaussview Version 6, 2019; Semichem Inc. Shawnee Mission KS.

[30] Breneman C. M., Wiberg K. B. (1990). Determining atom-centered
monopoles from molecular
electrostatic potentials. the need for high sampling density in formamide
conformational analysis. J. Comput. Chem..

[31] Martínez L., Andrade R., Birgin E. G., Martínez J. M. (2009). José
Mario Martínez. Packmol: A package for building initial configurations
for molecular dynamics simulations. J. Comput.
Chem..

[32] Jorgensen W. L., Maxwell D. S., Tirado-Rives J. (1996). Development and testing of the opls
all-atom force field on conformational energetics and properties of
organic liquids. J. Am. Chem. Soc..

[33] Haug E. J., Arora J. S., Matsui K. (1976). A steepest
descent method for optimization
of mechnical systems. J. Optim. Theor. Appl..

[34] Evans D. J., Holian B. L. (1985). The nose–hoover thermostat. J. Chem. Phys..

[35] Elber R., Ruymgaart A., Hess B., parallelization S. (2011). Eur. Phys. J.: Spec.
Top..

[36] Gajardo-Parra N.(á)s F., Cotroneo-Figueroa V. P., Aravena P., Vesovic V., Canales R. I. (2020). Viscosity
of choline chloride-based deep eutectic solvents:
Experiments and modeling. J. Chem. Eng. Data.

[37] Panda P., Mishra S. (2023). Deep eutectic solvents:
Physico-chemical properties
and their use for recovery of metal values from waste products. J. Mol. Liq..

[38] Yang F., Zheng Q., Tan H., Wang X. (2024). Insight into the role
of hydrogen bond donor in deep eutectic solvents. J. Mol. Liq..

[39] Jiang B., Yu Qi-K., Zhou Y., Yu C.-F., Zhang Yi, Wang J. (2025). Study of the effects
of different head group structures of dodecyl
anionic surfactants on the wettability of bituminous coal: experiments
and molecular simulations. Process Saf. Environ.
Prot..

[40] Dawass N., Krüger P., Schnell S. K., Simon J.-M., Vlugt T. J. H. (2019). Kirkwood-buff
integrals from molecular simulation. Fluid Phase
Equilib..

[41] Zhang J., Zhang P.Y., Ma K., Han F., Chen G.H., Wei X. (2008). Hydrogen bonding interactions between
ethylene glycol and water:
Density, excess molar volume, and spectral study. Sci. China, Ser. B:Chem..

[42] Wang Y., Wang Z., Liu Z., He X., Zeng Z. (2025). Mechanistic
insights into m-cresol adsorption on functional resins: Surface chemistry
and adsorption behavior. Materials.

[43] Zhang Q., De Oliveira Vigier K., Royer S., Jérôme F. (2012). Deep eutectic
solvents: syntheses, properties and applications. Chem. Soc. Rev..

[44] Contreras-García J., Yang W., Johnson E. R. (2011). Analysis of hydrogen-bond interaction
potentials from the electron density: Integration of noncovalent interaction
regions. J. Phys. Chem. A.

[45] Behera S. K., Gupta A. K., Rai G. (2025). Influence of li+ and na+ metal cations
on the structure and dynamics of 1-ethyl-3-methylimidazolium trifluoromethanesulfonate
[emim] [otf] ionic liquid- a molecular dynamics simulations study. J. Mol. Liq..

[46] Gupta A. K. (2022). Sr2+ and
ba2+ salts induced conformational structure of sodium polyacrylate
paa investigated by molecular dynamics simulations. Comput. Mater. Sci..

[47] Nettleton R. E., Green M. S. (1958). Expression for the
entropy of a system of interacting
particles. J. Chem. Phys..

[48] Ghaffarizadeh S. A., Wang G. J. (2024). A picture is worth a thousand timesteps: Excess entropy
scaling for rapid estimation of diffusion coefficients in molecular-dynamics
simulations of fluids. J. Chem. Theory Comput..

