# Supplementary material for: Molecular Simulations Investigation of Deep Eutectic Solvent Induced Structural and Interfacial Behavior in Surfactant-Enhanced Oil Recovery Systems
Source: ACS Omega. 2026 Oct 1;11(40):61419–33. doi: 10.1021/acsomega.6c06556 (PMC13670872; doi:10.1021/acsomega.6c06556)
Supplement: Supplementary file 1 [file ao6c06556_si_001.pdf]

Supporting Information

Molecular Simulations Investigation of Deep Eutectic Solvent (DES) induced Structural and Interfacial behaviour in Surfactant EOR systems

Subodh Sinha[1] and Abhishek Kumar Gupta[2,*]

[1,2]Department of Chemical Engineering, Indian Institute of Technology Kharagpur, Kharagpur, West Bengal 721302, India

*Correspondence to: Abhishek Kumar Gupta, Department of Chemical Engineering, Indian Institute of Technology Kharagpur, Kharagpur, West Bengal 721302, India.Tel: (91)–03222–214586.
*Corresponding Author: E–mail:akg@che.iitkgp.ac.in

Force Field Parameters for Choline Chloride : Cresol (ChCl:Cresol)

This section reports the complete force field parameters used for the ChCl:cresol system, as implemented in LAMMPS, including Lennard–Jones (pair_coeff), bond (bond_coeff), angle (angle_coeff), and dihedral (dihedral_coeff) parameters.

Table S1. Lennard–Jones parameters (pair_coeff) for ChCl:cresol.

Type i	Type j	ε (kcal/mol)	σ (Å)	Atom label
1	1	0.066000	3.5000000	C1
2	2	0.066000	3.5000000	C2
3	3	0.070000	3.5500000	C3
4	4	0.066000	3.5000000	C4
5	5	0.066000	3.5000000	C5
6	6	0.070000	3.5500000	C6
7	7	0.066000	3.5000000	C7
8	8	0.117790	4.1800000	CL
9	9	0.000000	0.0000000	WATER H
10	10	0.030000	2.5000000	H1
11	11	0.030000	2.5000000	H2
12	12	0.030000	2.4200000	H3
13	13	0.030000	0.5000000	H4
14	14	0.030000	2.5000000	H5
15	15	0.030000	2.5000000	H6
16	16	0.030000	2.4200000	H7
17	17	0.170000	3.2500000	N
18	18	0.002772	3.3500000	NA
19	19	0.155300	3.1660000	O
20	20	0.170000	3.1200000	O1
21	21	0.170000	3.1200000	O2
22	22	0.140000	2.9000000	O3
23	23	0.250000	3.5500000	S

Table S2. Bond parameters (bond_coeff) for ChCl:cresol.

Bond ID	k (kcal/mol/Å^2)	r_0 (Å)	Comment
1	268.0000	1.5290	C1–C1
2	320.0000	1.4100	C1–H1
3	367.0000	1.4710	C1–N
4	320.0000	1.4100	C1–O1
5	268.0000	1.5290	C2–C2
6	340.0000	1.0900	C2–H2
7	469.0000	1.4000	C3–C3
8	317.0000	1.5100	C3–C7
9	367.0000	1.0800	C3–H3
10	450.0000	1.3640	C3–O2
11	268.0000	1.5290	C4–C4
12	340.0000	1.0900	C4–H5
13	268.0000	1.5290	C5–C5
14	268.0000	1.5290	C5–C6

Bond ID	k (kcal/mol/Å^2)	r_0 (Å)	Comment
15	340.0000	1.0900	C5–H6
16	469.0000	1.4000	C6–C6
17	367.0000	1.0800	C6–H7
18	340.0000	1.7700	C6–S
19	340.0000	1.0900	C7–H3
20	450.0000	1.0000	H–O
21	553.0000	0.9450	H1–O1
22	553.0000	0.9450	H4–O2
23	700.0000	1.4400	O3–S

Table S3. Angle parameters (angle_coeff) for ChCl:cresol.

Angle ID	k (kcal/mol/rad^2)	θ_0 (deg)	Comment
1	37.500	110.700	C1–C1–H1
2	80.000	111.200	C1–C1–N
3	50.000	109.500	C1–C1–O1
4	50.000	113.000	C1–N–C1
5	55.000	108.500	C1–O1–H1
6	58.350	112.700	C2–C2–C2
7	37.500	110.700	C2–C2–H2
8	63.000	120.000	C3–C3–C3
9	70.000	120.000	C3–C3–C7
10	35.000	120.000	C3–C3–H3
11	70.000	120.000	C3–C3–O2
12	35.000	109.500	C3–C7–H3
13	35.000	113.000	C3–O2–H4
14	58.350	112.700	C4–C4–C4
15	37.500	110.700	C4–C4–H5
16	58.350	112.700	C5–C5–C5
17	58.350	112.700	C5–C5–C6
18	37.500	110.700	C5–C5–H6
19	70.000	120.000	C5–C6–C6
20	37.500	110.700	C6–C5–H6
21	63.000	120.000	C6–C6–C6
22	55.000	108.500	C6–C6–H7
23	85.000	119.400	C6–C6–S
24	78.670	104.360	C6–S–O3
25	55.000	109.500	H–O–H
26	33.000	107.800	H1–C1–H1
27	35.000	109.500	H1–C1–N
28	35.000	109.500	H1–C1–O1
29	33.000	107.800	H2–C2–H2
30	33.000	107.800	H3–C7–H3
31	33.000	107.800	H5–C4–H5
32	33.000	107.800	H6–C5–H6
33	78.670	104.360	O3–S–O3

Table S4. Dihedral parameters (dihedral_coeff) for ChCl:cresol.

Dihedral ID	K_1	K_2	K_3	K_4	Comment
1	1.438	-0.124	0.264	0.000	C1–C1–N–C1
2	-0.356	-0.174	0.492	0.000	C1–C1–O1–H1
3	1.300	-0.200	0.200	0.000	C2–C2–C2–C2
4	0.000	0.000	0.300	0.000	C2–C2–C2–H2
5	0.000	7.250	0.000	0.000	C3–C3–C3–C3
6	0.000	7.250	0.000	0.000	C3–C3–C3–C7
7	0.000	7.250	0.000	0.000	C3–C3–C3–H3
8	0.000	7.250	0.000	0.000	C3–C3–C3–O2
9	0.000	0.000	0.000	0.000	C3–C3–C7–H3
10	0.000	1.682	0.000	0.000	C3–C3–O2–H4
11	1.300	-0.200	0.200	0.000	C4–C4–C4–C4
12	0.000	0.000	0.300	0.000	C4–C4–C4–H5
13	1.300	-0.200	0.200	0.000	C5–C5–C5–C5
14	1.300	-0.200	0.200	0.000	C5–C5–C5–C6
15	0.000	0.000	0.300	0.000	C5–C5–C5–H6
16	1.300	-0.200	0.200	0.000	C5–C5–C6–C6
17	1.300	-0.200	0.200	0.000	C5–C6–C6–C6
18	0.000	0.000	0.300	0.000	C5–C6–C6–H7
19	0.000	7.250	0.000	0.000	C5–C6–C6–S
20	0.000	0.000	0.300	0.000	C6–C5–C5–H6
21	1.300	-0.200	0.200	0.000	C6–C5–C5–C6
22	0.000	0.000	0.300	0.000	C6–C5–C5–H7
23	0.000	7.250	0.000	0.000	C6–C6–C6–S
24	0.000	0.000	0.000	0.000	C6–C6–S–O3
25	0.000	7.250	0.000	0.000	C7–C3–C3–H3
26	0.000	0.000	0.300	0.000	H1–C1–C1–H1
27	0.000	0.000	0.384	0.000	H1–C1–C1–N
28	0.000	0.000	0.468	0.000	H1–C1–C1–O1
29	0.000	0.000	0.302	0.000	H1–C1–N–C1
30	0.000	0.000	0.352	0.000	H1–C1–O1–H1
31	0.000	0.000	0.300	0.000	H2–C2–C2–H2
32	0.000	7.250	0.000	0.000	H3–C3–C3–H3
33	0.000	7.250	0.000	0.000	H3–C3–C3–O2
34	0.000	0.000	0.300	0.000	H5–C4–C4–H5
35	0.000	0.000	0.300	0.000	H6–C5–C5–H6
36	0.000	0.000	0.300	0.000	H6–C5–C5–C6
37	0.000	0.000	0.300	0.000	H7–C6–C6–H7
38	0.000	0.000	0.300	0.000	H7–C6–C6–S
39	8.000	0.000	0.000	0.000	N–C1–C1–O1

Force Field Parameters for Choline Chloride : Ethylene Glycol (ChCl:EG)

This section reports the complete force field parameters used for the ChCl:EG system, as implemented in LAMMPS, including Lennard–Jones (pair_coeff), bond (bond_coeff), angle (angle_coeff), and dihedral (dihedral_coeff) parameters.

Table S5. Lennard–Jones parameters (pair_coeff) for ChCl:EG.

Type i	Type j	ε (kcal/mol)	σ (Å)	Atom label
1	1	0.066000	3.5000000	C1
2	2	0.066000	3.5000000	C2
3	3	0.066000	3.5000000	C3
4	4	0.066000	3.5000000	C4
5	5	0.066000	3.5000000	C5
6	6	0.070000	3.5500000	C6
7	7	0.117790	4.1800000	CL
8	8	0.000000	0.0000000	WATER H
9	9	0.030000	2.5000000	H1
10	10	0.030000	2.5000000	H2
11	11	0.030000	2.5000000	H3
12	12	0.000000	0.0000000	H4
13	13	0.030000	2.5000000	H5
14	14	0.030000	2.5000000	H6
15	15	0.030000	2.4200000	H7
16	16	0.170000	3.2500000	N
17	17	0.002772	3.3500000	NA
18	18	0.155300	3.1660000	O
19	19	0.170000	3.1200000	O1
20	20	0.170000	3.1200000	O2
21	21	0.140000	2.9000000	O3
22	22	0.250000	3.5500000	S

Table S6. Bond parameters (bond_coeff) for ChCl:EG.

Bond ID	k (kcal/mol/Å^2)	r_0 (Å)	Comment
1	268.0000	1.5290	C1–C1
2	320.0000	1.4100	C1–H1
3	367.0000	1.4710	C1–N
4	320.0000	1.4100	C1–O1
5	268.0000	1.5290	C2–C2
6	340.0000	1.0900	C2–H2
7	268.0000	1.5290	C3–C3
8	340.0000	1.0900	C3–H3
9	320.0000	1.4100	C3–O2
10	268.0000	1.5290	C4–C4
11	340.0000	1.0900	C4–H5
12	268.0000	1.5290	C5–C5
13	268.0000	1.5290	C5–C6
14	340.0000	1.0900	C5–H6
15	469.0000	1.4000	C6–C6
16	367.0000	1.0800	C6–H7
17	340.0000	1.7700	C6–S
18	450.0000	1.0000	H–O
19	553.0000	0.9450	H1–O1
20	553.0000	0.9450	H4–O2

Bond ID	k (kcal/mol/Å^2)	r_0 (Å)	Comment
21	700.0000	1.4400	O3–S

Table S7. Angle parameters (angle_coeff) for ChCl:EG.

Angle ID	k (kcal/mol/rad^2)	θ_0 (deg)	Comment
1	37.500	110.700	C1–C1–H1
2	80.000	111.200	C1–C1–N
3	50.000	109.500	C1–C1–O1
4	50.000	113.000	C1–N–C1
5	55.000	108.500	C1–O1–H1
6	58.350	112.700	C2–C2–C2
7	37.500	110.700	C2–C2–H2
8	37.500	110.700	C3–C3–H3
9	50.000	109.500	C3–C3–O2
10	55.000	108.500	C3–O2–H4
11	58.350	112.700	C4–C4–C4
12	37.500	110.700	C4–C4–H5
13	58.350	112.700	C5–C5–C5
14	58.350	112.700	C5–C5–C6
15	37.500	110.700	C5–C5–H6
16	70.000	120.000	C5–C6–C6
17	37.500	110.700	C6–C5–H6
18	63.000	120.000	C6–C6–C6
19	55.000	108.500	C6–C6–H7
20	85.000	119.400	C6–C6–S
21	78.670	104.360	C6–S–O3
22	55.000	109.500	H–O–H
23	33.000	107.800	H1–C1–H1
24	35.000	109.500	H1–C1–N
25	35.000	109.500	H1–C1–O1
26	33.000	107.800	H2–C2–H2
27	33.000	107.800	H3–C3–H3
28	35.000	109.500	H3–C3–O2
29	33.000	107.800	H5–C4–H5
30	33.000	107.800	H6–C5–H6
31	78.670	104.360	O3–S–O3

Table S8. Dihedral parameters (dihedral_coeff) for ChCl:EG.

Dihedral ID	K_1	K_2	K_3	K_4	Comment
1	1.438	-0.124	0.264	0.000	C1–C1–N–C1
2	-0.356	-0.174	0.492	0.000	C1–C1–O1–H1
3	1.300	-0.200	0.200	0.000	C2–C2–C2–C2
4	0.000	0.000	0.300	0.000	C2–C2–C2–H2
5	-0.356	-0.174	0.492	0.000	C3–C3–O2–H4
6	1.300	-0.200	0.200	0.000	C4–C4–C4–C4
7	0.000	0.000	0.300	0.000	C4–C4–C4–H5

Dihedral ID	K_1	K_2	K_3	K_4	Comment
8	1.300	-0.200	0.200	0.000	C5–C5–C5–C5
9	1.300	-0.200	0.200	0.000	C5–C5–C5–C6
10	0.000	0.000	0.300	0.000	C5–C5–C5–H6
11	1.300	-0.200	0.200	0.000	C5–C5–C6–C6
12	1.300	-0.200	0.200	0.000	C5–C6–C6–C6
13	0.000	0.000	0.300	0.000	C5–C6–C6–H7
14	0.000	7.250	0.000	0.000	C5–C6–C6–S
15	0.000	0.000	0.300	0.000	C6–C5–C5–H6
16	1.300	-0.200	0.200	0.000	C6–C5–C5–C6
17	0.000	0.000	0.300	0.000	C6–C5–C5–H7
18	0.000	7.250	0.000	0.000	C6–C6–C6–S
19	0.000	0.000	0.000	0.000	C6–C6–S–O3
20	0.000	0.000	0.300	0.000	H1–C1–C1–H1
21	0.000	0.000	0.384	0.000	H1–C1–C1–N
22	0.000	0.000	0.468	0.000	H1–C1–C1–O1
23	0.000	0.000	0.302	0.000	H1–C1–N–C1
24	0.000	0.000	0.352	0.000	H1–C1–O1–H1
25	0.000	0.000	0.300	0.000	H2–C2–C2–H2
26	0.000	0.000	0.300	0.000	H3–C3–C3–H3
27	0.000	0.000	0.468	0.000	H3–C3–C3–O2
28	0.000	0.000	0.352	0.000	H3–C3–O2–H4
29	0.000	0.000	0.300	0.000	H5–C4–C4–H5
30	0.000	0.000	0.300	0.000	H6–C5–C5–H6
31	0.000	0.000	0.300	0.000	H6–C5–C5–C6
32	0.000	0.000	0.300	0.000	H7–C6–C6–H7
33	0.000	0.000	0.300	0.000	H7–C6–C6–S
34	8.000	0.000	0.000	0.000	N–C1–C1–O1
35	9.508	0.000	0.000	0.000	O2–C3–C3–O2

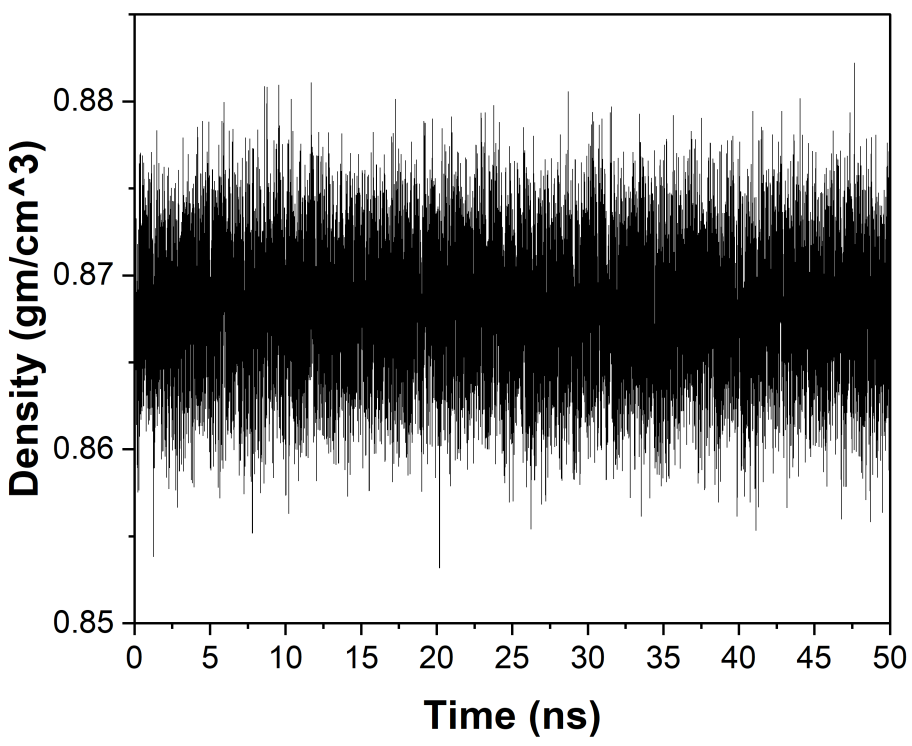

(a) Density, ChCl:EG at 15 wt%

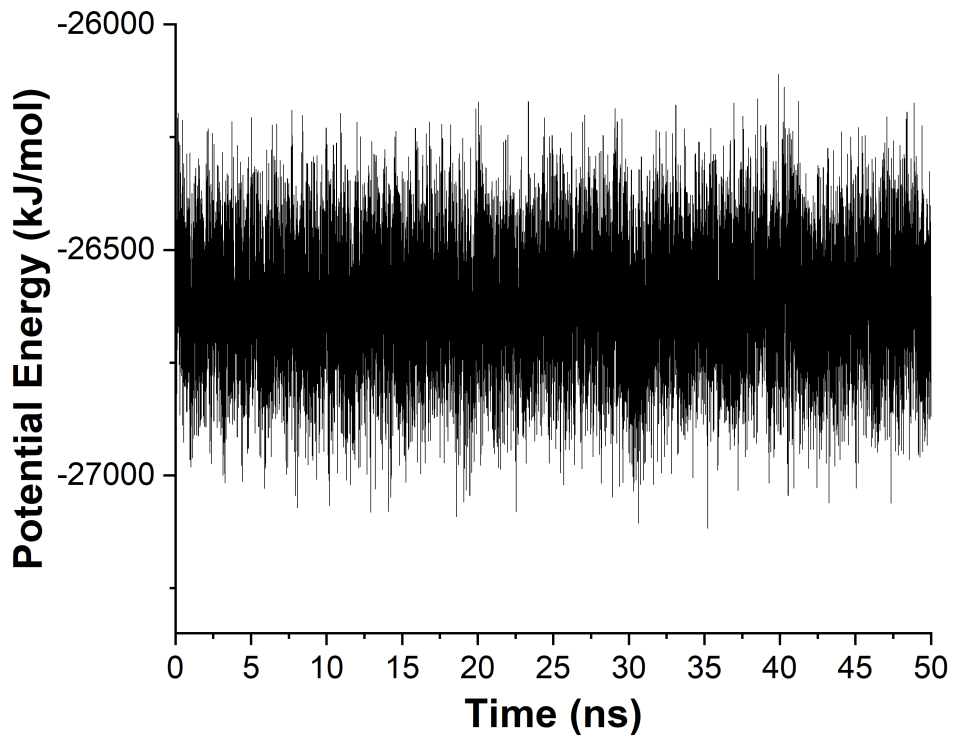

(b) Potential energy, ChCl:EG at 15 wt%

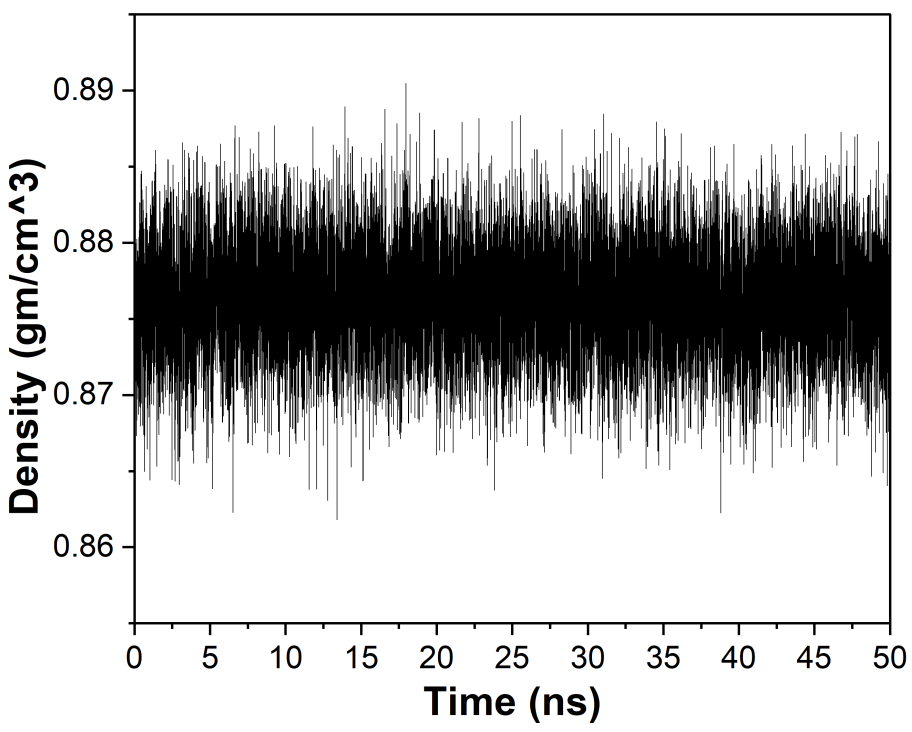

(c) Density, ChCl:EG at 25 wt%

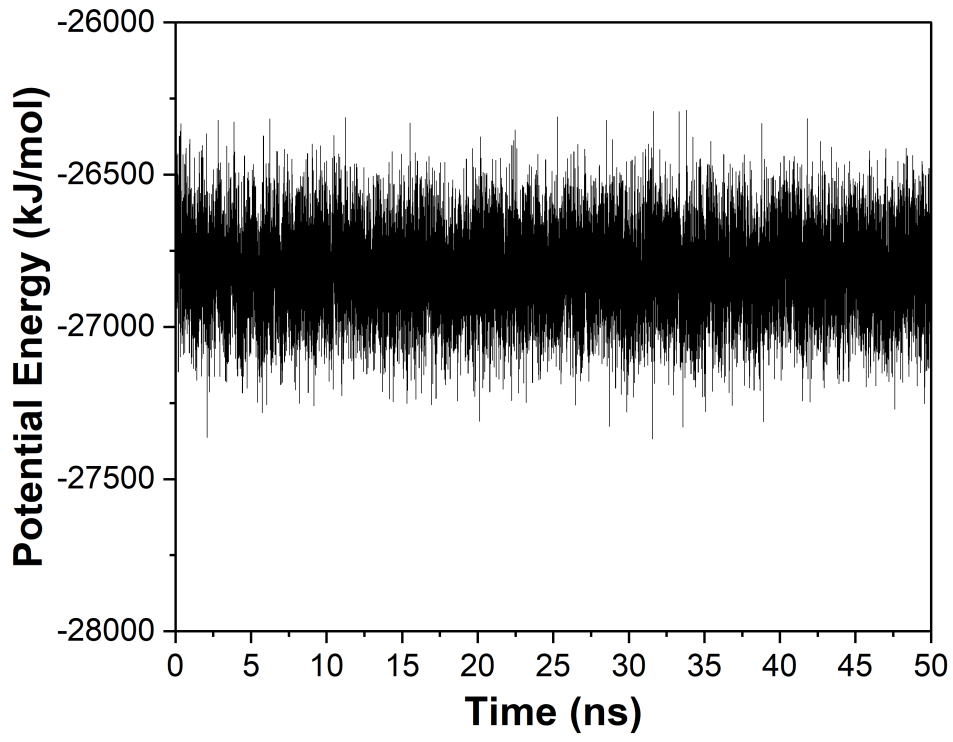

(d) Potential energy, ChCl:EG at 25 wt%

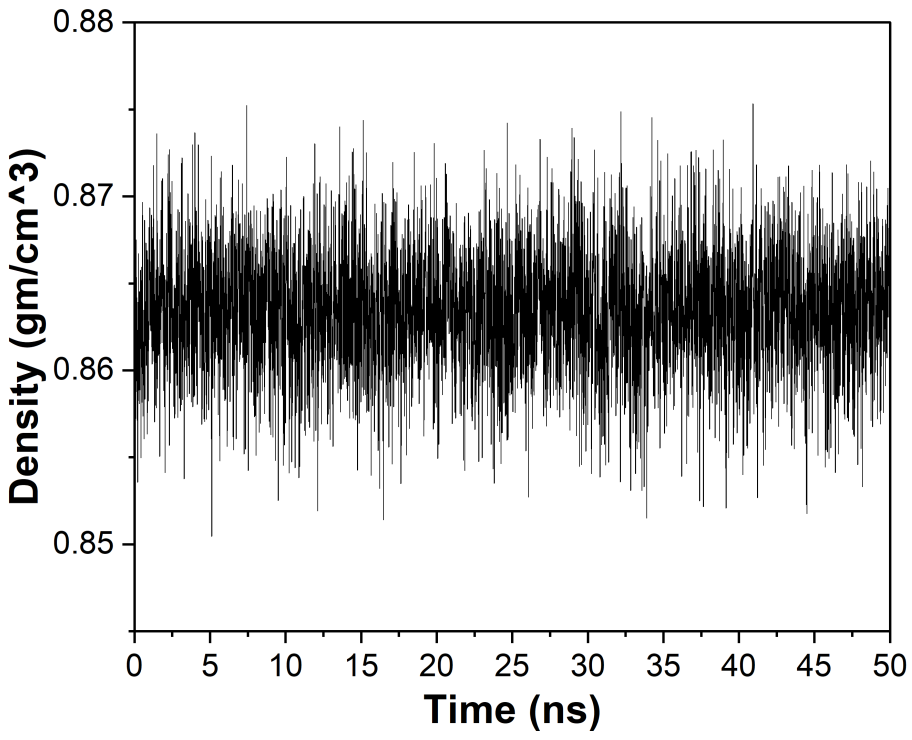

(e) Density, ChCl:Cresol at 10 wt%

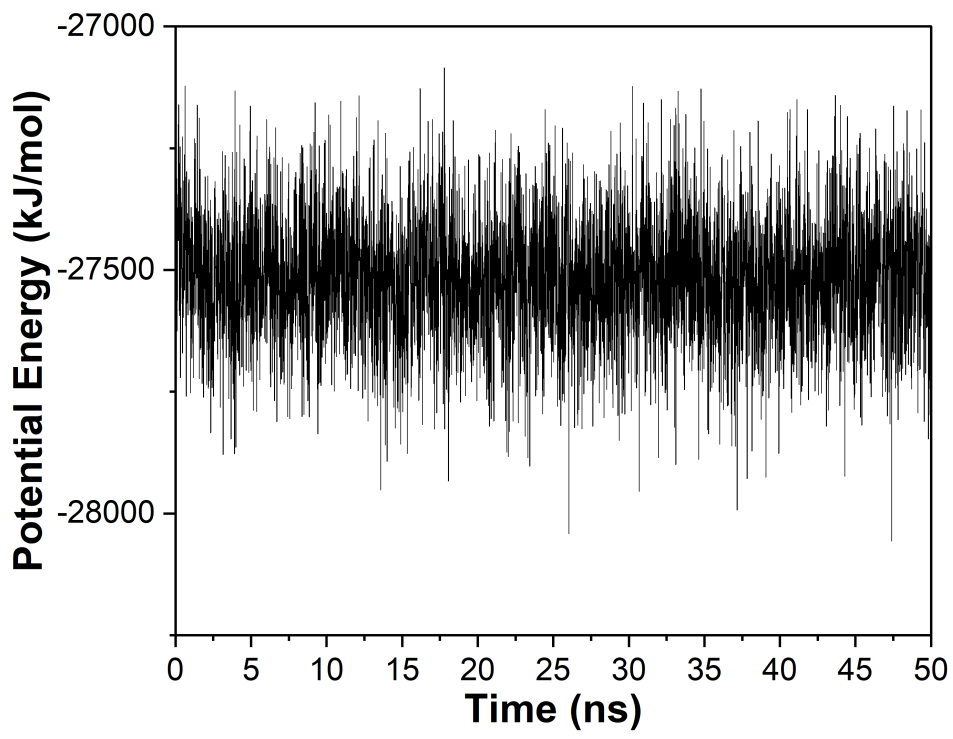

(f) Potential energy, ChCl:Cresol at 10 wt%

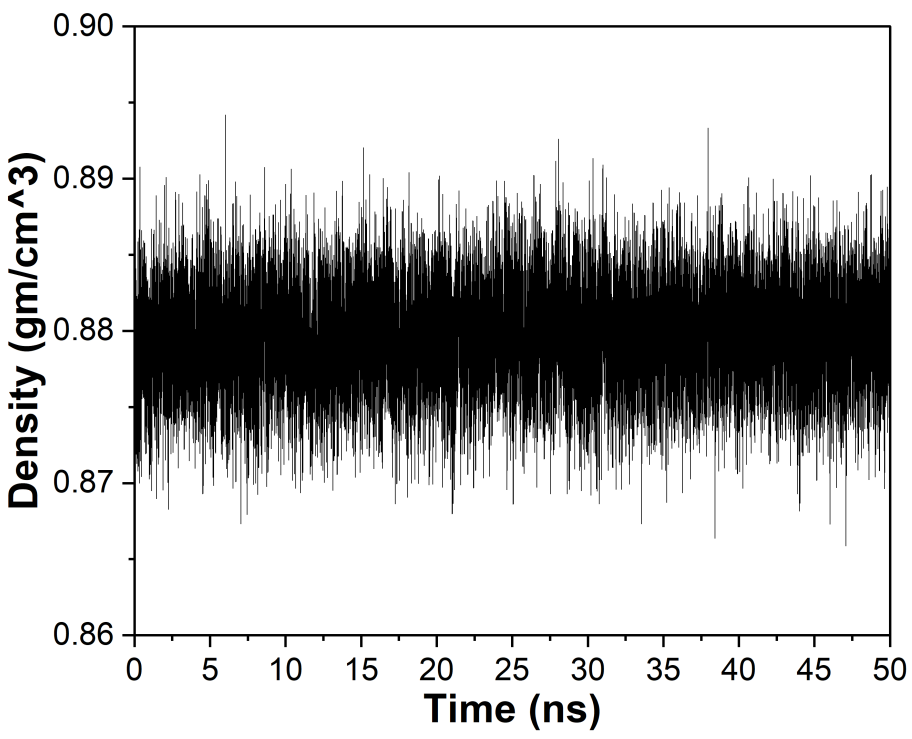

(g) Density, ChCl:Cresol at 30 wt%

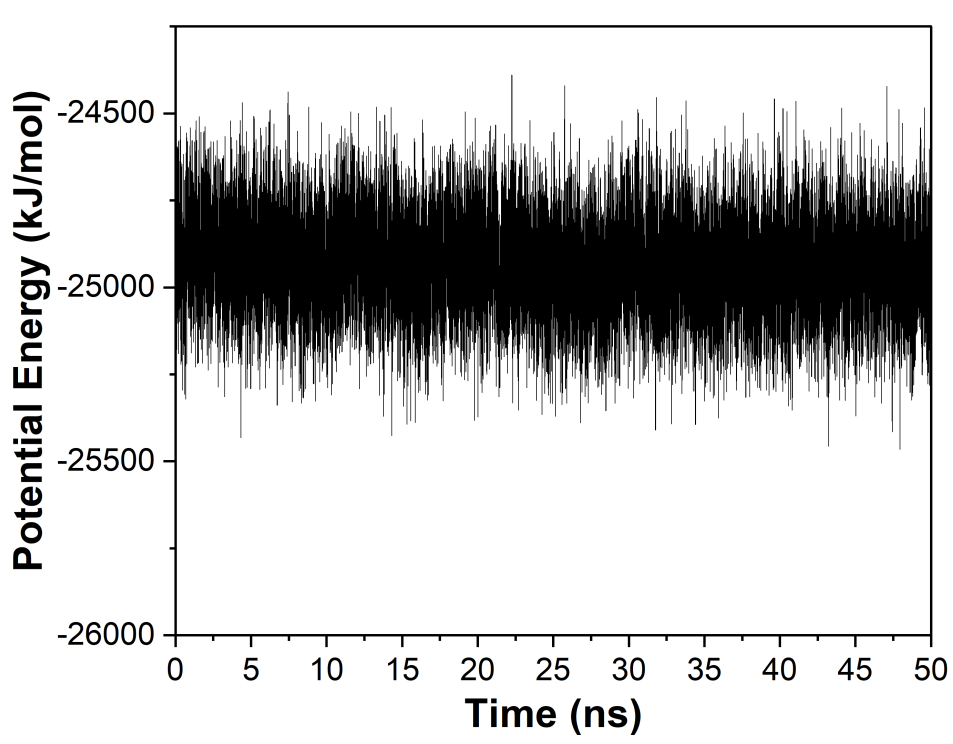

(h) Potential energy, ChCl:Cresol at 30 wt%

Figure S1: Equilibration curves showcasing system density and potential energy evolution with time of the simulated ChCl:EG and ChCl:Cresol systems at different DES concentrations.

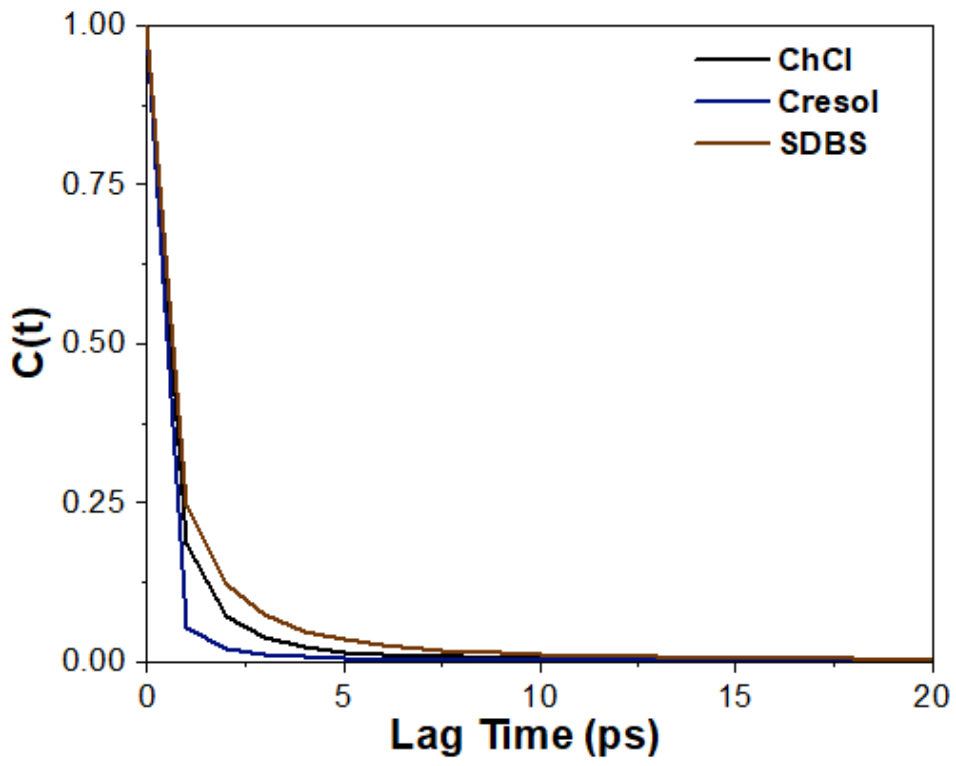

(a) ChCl + Cresol, 10 wt%

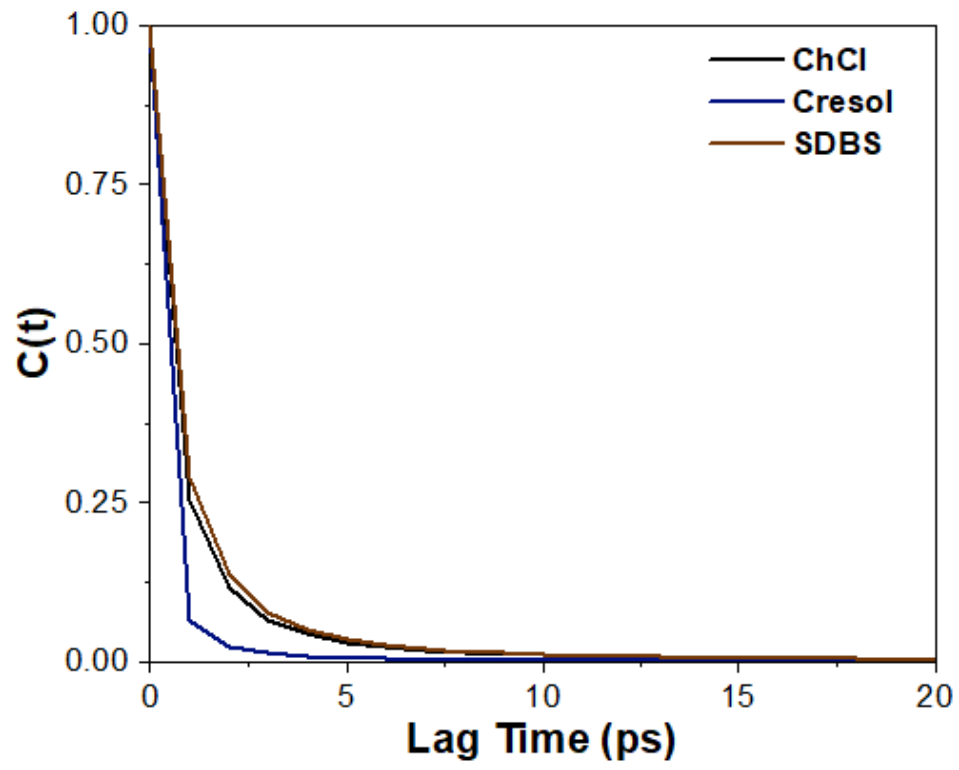

(b) ChCl + Cresol, 30 wt%

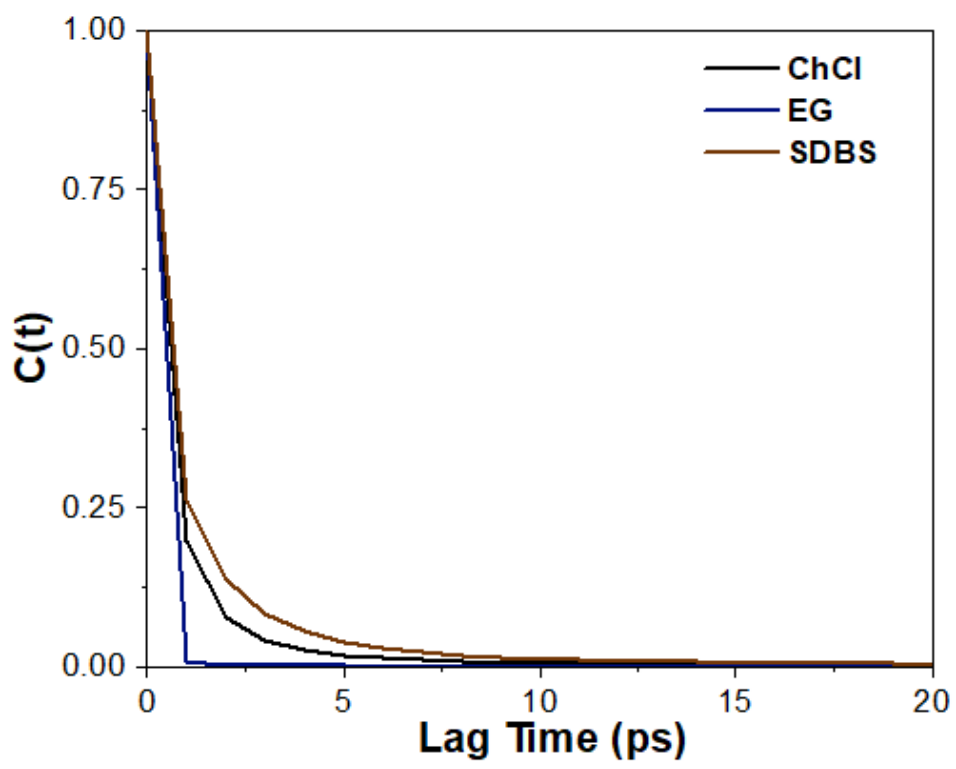

(c) ChCl + EG, 10 wt%

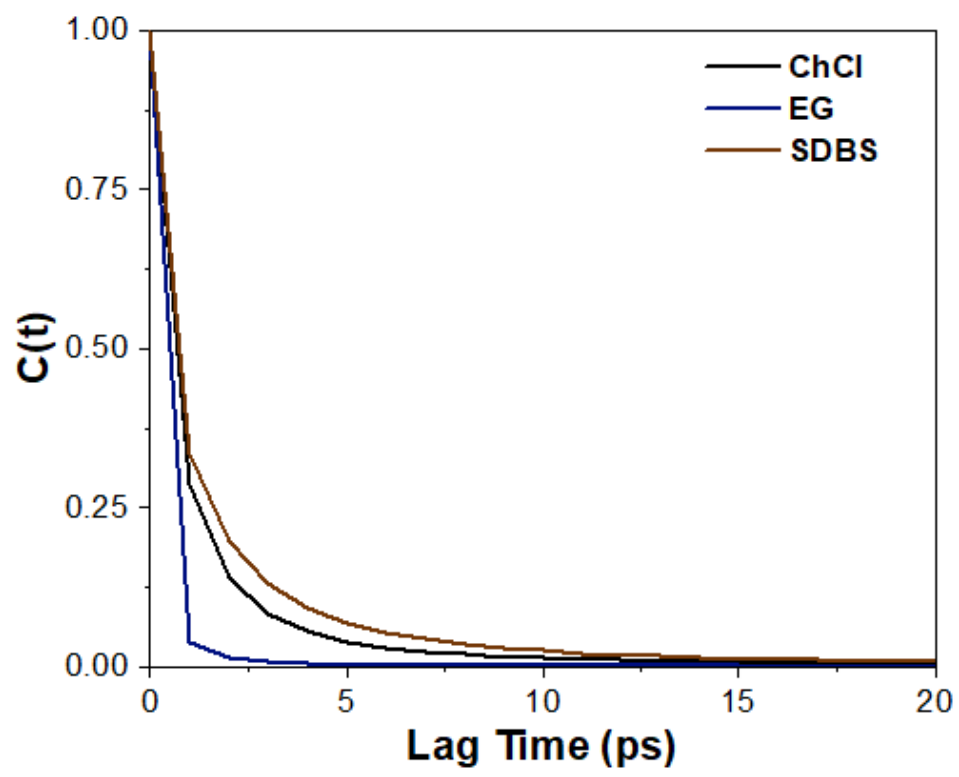

(d) ChCl + EG, 30 wt%

Figure S2: Hydrogen-bond autocorrelation functions for the ChCl-based DES systems at 10 and 30 wt% concentrations.
